# Recent advances in optical imaging through deep tissue: imaging probes and techniques

**DOI:** 10.1186/s40824-022-00303-4

**Published:** 2022-10-22

**Authors:** Seokchan Yoon, Seo Young Cheon, Sangjun Park, Donghyun Lee, Yeeun Lee, Seokyoung Han, Moonseok Kim, Heebeom Koo

**Affiliations:** 1 School of Biomedical Convergence Engineering, Pusan National University, Yangsan, 50612 Republic of Korea; 2 Department of Medical Life Sciences and Department of Biomedicine & Health Sciences, College of Medicine, The Catholic University of Korea, Seoul, 06591 Republic of Korea; 3 Department of Mechanical Engineering, University of Louisville, Louisville, KY 40208 USA; 4 Catholic Photomedicine Research Institute, College of Medicine, The Catholic University of Korea, Seoul, 06591 Republic of Korea

**Keywords:** Optical imaging, Deep tissue imaging, Imaging probe, NIR-II, Bioluminescence, Chemiluminescence, Adaptive optics, Wavefront sensing, Wavefront shaping

## Abstract

Optical imaging has been essential for scientific observations to date, however its biomedical applications has been restricted due to its poor penetration through tissues. In living tissue, signal attenuation and limited imaging depth caused by the wave distortion occur because of scattering and absorption of light by various molecules including hemoglobin, pigments, and water. To overcome this, methodologies have been proposed in the various fields, which can be mainly categorized into two stategies: developing new imaging probes and optical techniques. For example, imaging probes with long wavelength like NIR-II region are advantageous in tissue penetration. Bioluminescence and chemiluminescence can generate light without excitation, minimizing background signals. Afterglow imaging also has high a signal-to-background ratio because excitation light is off during imaging. Methodologies of adaptive optics (AO) and studies of complex media have been established and have produced various techniques such as direct wavefront sensing to rapidly measure and correct the wave distortion and indirect wavefront sensing involving modal and zonal methods to correct complex aberrations. Matrix-based approaches have been used to correct the high-order optical modes by numerical post-processing without any hardware feedback. These newly developed imaging probes and optical techniques enable successful optical imaging through deep tissue. In this review, we discuss recent advances for multi-scale optical imaging within deep tissue, which can provide reseachers multi-disciplinary understanding and broad perspectives in diverse fields including biophotonics for the purpose of translational medicine and convergence science.

**Graphical Abstract:**

Methodologies for multi-scale optical imaging within deep tissues are discussed in diverse fields including biophotonics for the purpose of translational medicine and convergence science. Recent imaging probes have tried deep tissue imaging by NIR-II imaging, bioluminescence, chemiluminescence, and afterglow imaging. Optical techniques including direct/indirect and coherence-gated wavefront sensing also can increase imaging depth.
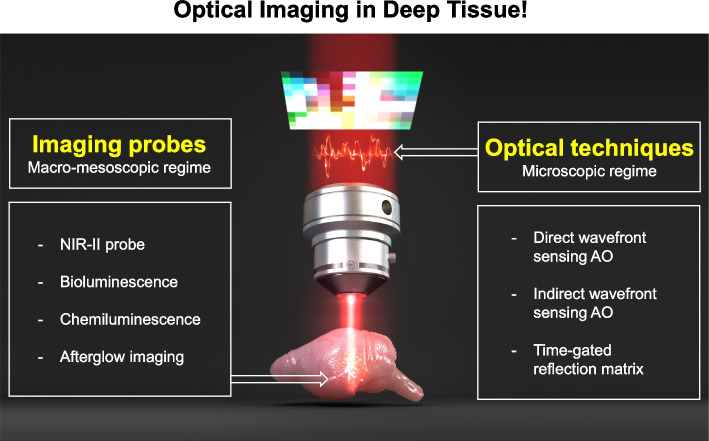

## Introduction

Like the proverb ‘seeing is believing’, an exact and precise diagnosis of a disease site is essential to curing the disease. For this purpose, various imaging modalities have been developed and used to date [1]. They include optical imaging, magnetic resonance imaging, computed tomography, ultrasound, single-photon emission computed tomography, and positron emission tomography [2]. Among them, optical imaging has been studied extensively because it is advantageous for the investigation of various physical and biological systems. Specifically, it can provide precise subcellular and molecular information with high resolution at the wavelength level with minimal hazard. However, the imaging depth accessible by optical imaging is shallow, and the spatial resolution rapidly degrades along with the axial depth since the propagation of light waves significantly deteriorates within the biological tissues by specimen-induced aberrations and multiple light scattering [3, 4]. Therefore, signal attenuation and limitations in imaging depth caused by wave distortion have been important issues in diverse fields of biophotonics over the past decades. These challenges have prevented the broad application of optical imaging in clinical practice.

Light in living tissue is scattered and absorbed extensively by various molecules including hemoglobin, pigments, and water, so that the intensity of the irradiated light severely decreases after passing through thick tissue [5]. A profound understanding of wave propagation in tissue is essential to interpreting the signal of single-scattered waves (referred to as ballistic waves), which include object information [6]. The signal and the point spread function (PSF) of single-scattered waves for epi-detection configurations can be physically described by ${\eta e}^{-2z/{l}_{\textrm{s}}}$, from which we can determine the two major origins of the signal attenuation. Here, *η* is the attenuation factor due to the aberrations, *l*_s_ is the scattering mean free path, and *z* is the imaging depth. The exponential term ${e}^{-2z/{l}_{\textrm{s}}}$ results from wave diffusion by multiple scattering. From this, the signal strength is reduced to only 13.5% at the depth of the scattering mean free path, which is on the order of hundreds of microns in biological tissues [7]. In addition to multiple scattering, the attenuation factor *η* by the sample-induced aberration significantly reduces the signal of the ballistic wave forming the PSF.

Therefore, attempts to improve the signal strength and signal-to-background ratio can be interpreted and classified into two major categories. One method is to shorten the ratio of the relative optical path 2*z*/*l*_s_. For instance, development of the NIR-II fluorescence-based fluorophore is useful for this using the light source of the wavelength that has a longer scattering mean free path. Exploitation of bioluminescence, chemiluminescence, and afterglow imaging helps eliminate the effect of the wave diffusion for the input path using the light emitted from the target itself within the tissue. The second method is to remove the attenuation factor by wave distortion at the given wavelength. Most methodologies of adaptive optics and studies of complex media have sought to resolve the attenuation of the ballistic wave by correcting the wave distortion. In this review, we describe those two major streams of the deep-tissue imaging from the perspective of the imaging probes and the optical techniques, which are approaches embracing multi-scale imaging corresponding to macro-mesoscopic and microscopic regimes, respectively. We will talk about the strategies and mechanisms of these trials and focus on their demonstration in tissues or animals, not just in cell experiments.

## Imaging probes for optical imaging through deep tissue

Imaging probes that can generate or enhance the imaging signals play pivotal role in imaging [8]. They are based on small molecule dyes, proteins, or inorganic nanoparticles. Chemical dyes have low molecular weights and defined structures, which are advantageous for mass production and quality control. Proteins can be produced by living organisms and (like biosimilar drugs) are linked to specific proteins by genetic engineering. Inorganic nanoparticles have their own special properties based on their components and micro-structures, and these properties are useful for imaging or therapy [9]. Researchers have used these imaging probes for optical imaging, and recently developed probes showed the potential to overcome the hurdles in deep tissue imaging (Table 1).

**Table 1:** Summary of the imaging probes for deep tissue imaging

Strategy	Advantages/obstacles	Material	In vivo application	Ref.
NIR-II	Deeper penetration depth compared to another wavelength region/Hard to develop hydrophilic NIR-II dyes.	Tat peptide-modified Pb/S quantum dot	Mesenchymal stem cell labelling and subcutaneous injection (mice).	[10]
		Heptamethine-cyanine-based NIR-II fluorophore	Intravenous injection and tumor imaging (mice).	[11]
		Metal nanotube-type phosphorescence probe	Subcutaneous injection and tumor imaging (mice)	[12]
Bioluminescence	No excitation/Need luciferase protein with low in vivo stability, Time-dependent change of imaging signals.	Nano-luciferase complex and diphenyltetrazine pair	Intravenous injection of plasmid and intraperitoneal injection of diphenyltetrazine substrate for red-shifted bioluminescence (mice).	[13]
		Red fluorescence protein-luciferase fusion protein and biothiol-responsive coelenterazine	Subcutaneous injection of biothiol-responsive coelenterazine and tumor imaging (mice).	[14]
		H-ferritin nanocage-luciferin conjugate	Intravenous injection of nanocage and tumor imaging (mice).	[15]
Chemiluminescence	No excitation/Time-dependent change of imaging signals.	Granzyme B-responsive phenoxydioxetane probe	Intratumoral injection of probe and imaging of NK cell effect in tumor (mice).	[16]
		Chemical conjugates of three components (NIRF dye, chemiluminescence dye, and cyclodextrin derivative).	Intravenous injection of the probe for dual imaging of drug-induced acute kidney injury (mice).	[17]
		Tetraphenylethylene-phthalhydrazid-based nanoparticle	Intravenous injection of probe and singlet oxygen imaging in arthritis model (rat).	[18]
After glow	No excitation/Time-dependent change of imaging signals. Relatively short imaging time.	ZnSn_2_O_4_:Cr,Eu nanoparticle	Intravenous injection of the nanoparticle and tumor imaging (mice).	[19]
		Semiconducting polymer (phenylenevinylene) nanoparticle	Intravenous injection of the nanoparticle and lymph node/ tumor imaging (mice).	[20]
		Nanoparticle of three components (afterglow initiator, substrate, and semiconducting polymer).	Intravenous injection of the nanoparticle and tumor imaging (mice).	[21]

### NIR-II imaging

Visible light is only suitable for cell or thin sectioned-tissue levels, not for thick tissues. The longer wavelength NIR-I window (650–900 nm) has less interaction with tissues and has been studied by many researchers for in vivo imaging. Indocyanine green, an FDA-approved dye, is a representative example. NIR-I fluorescence-based fluorophores have been used for image-guided surgery through the classification of diseased and normal tissues [22]. However, the tissue-penetration depth of NIR-I light was less than 1 mm, which was still insufficient for deep tissue imaging. Recently, the NIR-II window with fewer tissue interactions has been studied, which includes the range of 1000–1700 nm and has a deeper penetration depth with lower light attenuation and reduced photon scattering compared to the NIR-I window [23]. Therefore, in vivo fluorescence imaging using the NIR-II window is suitable for deep tissue imaging resulting in a high signal-to-background ratio and low autofluorescence [24]. Despite these advantages, NIR-II probes are not widely used because of obstacles such as low quantum yield and poor water solubility. To solve this, semiconducting polymer nanoparticles, quantum dots (QDs), and new water-soluble small molecule probes are being studied as NIR-II agents.

**Scheme 1:**
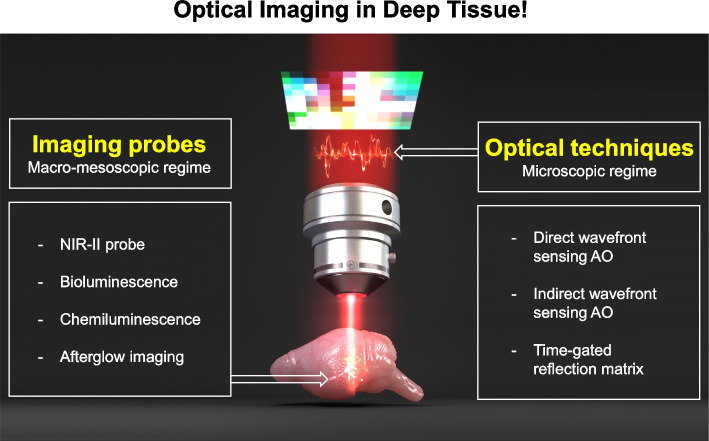
Schematic illustration of deep tissue imaging strategies using imaging probes and optical techniques

Yang et al. developed nanocrystal QDs that have fluorescence in the NIR-II window for tracking mesenchymal stem cells (MSC) [10]. The QDs were synthesized by reacting Pb and S with RNase-A as a protein template, and cell-penetrating tat peptide was conjugated to the QD surface to increase cellular uptake. When QDs were irradiated with an 808 nm laser, emission was observed at 1100 nm, which enabled cell tracking in deep tissue. The QDs did not affect the cell cycle, differentiation capacity, and viability of MSC at 10 to 30 μg/ml. Internalized quantum dots in MSCs were stably located in the cytoplasm in that their excretion was not significant in the trans-well plate for 7 days. When the QD-labeled MSCs were subcutaneously injected into mice, the detection limit was 1 × 10^3 cells, and a stable fluorescence signal was observed for 28 days. The previously reported CdSe@ZnS QDs emitting visible light QDs showed a higher detection limit (5 × 10^4 cells) compared to the developed QD, demonstrating the advantage of the NIR-II imaging agent for in vivo cell tracking. The QDs present in mice were slowly decomposed into Pb ions and excreted in urine and feces, and almost no signals were observed 42 days post-injection in organs except for the kidney, liver, and spleen (Scheme 1).

**Fig. 1:**
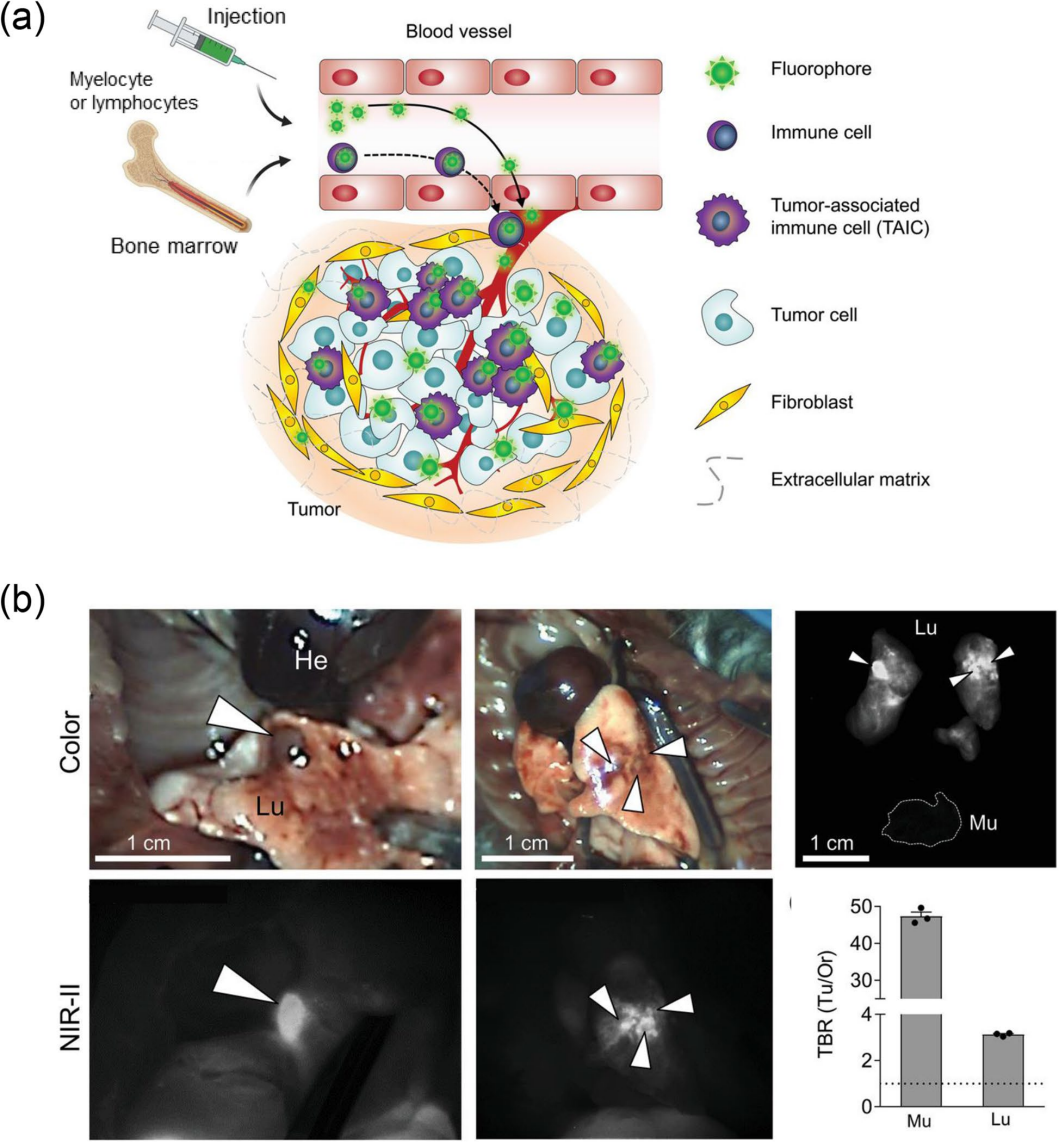
NIR-II fluorescent imaging probe for immune cell-mediated tumor imaging. **a** Schematic illustration of the strategy for tumor accumulation and imaging. **b** NIR-II imaging in tumor tissue using mice. Adapted with permission from Ref. [11]

Recently, Choi’s group reported SH1, a heptamethine-cyanine-based NIR-II fluorophore, without chemical conjugation of the targeting ligand for optical tumor imaging [11]. SH1 possessed maximal emission at 820 nm and the tail emission was up to about 1250 nm (Fig. 1a). They compared NIR-I and NIR-II fluorescence imaging using living tissue-mimicking gelatin phantoms to investigate the penetration depth. In NIR-II, SH1 showed 4.8-fold higher signal-to-background ratio than that in NIR-I for 12 mm thick tissue phantoms. Therefore, for in vivo experiments, NIR-II at 1070 nm was used due to its deeper penetration depth and lower autofluorescence than NIR-I. The quantum yield of SH1 was 11%, which was higher than that of commercialized NIR-II dyes (less than 2%). Fluorescence brightness was also 2.2-and 1.7-fold higher than indocyanine green and IR-780, respectively. To estimate the in vivo tumor-specific imaging performance, they performed NIR-II fluorescence imaging at various time points after intravenous injection of SH1, indocyanine green (ICG) or IR-780 into LLC tumor-bearing mice. ICG fluorescence signals in the tumor site were negligible. IR-780 showed a strong fluorescence signal in the tumor 4 hours post-injection, but the signal decreased after removing the skin around the tumor at 48 hours, and the signal was just observable in the tumor outline. On the other hand, SH1 showed a strong fluorescence signal in the tumor from 24 h, and the signal was maintained even when the skin was removed after 48 hours. This late tumor accumulation pattern was because SH1 targeted immune cells in the bone marrow and subsequently migrated with them. With this mechanism, the tumor-to-background ratio (TBR) was greater than 9 for tumors ranging from small 5 mm pancreatic cancer tumors (Pan02) to 15 mm large breast cancer tumors (E0771) (Fig. 1b).

**Fig. 2:**
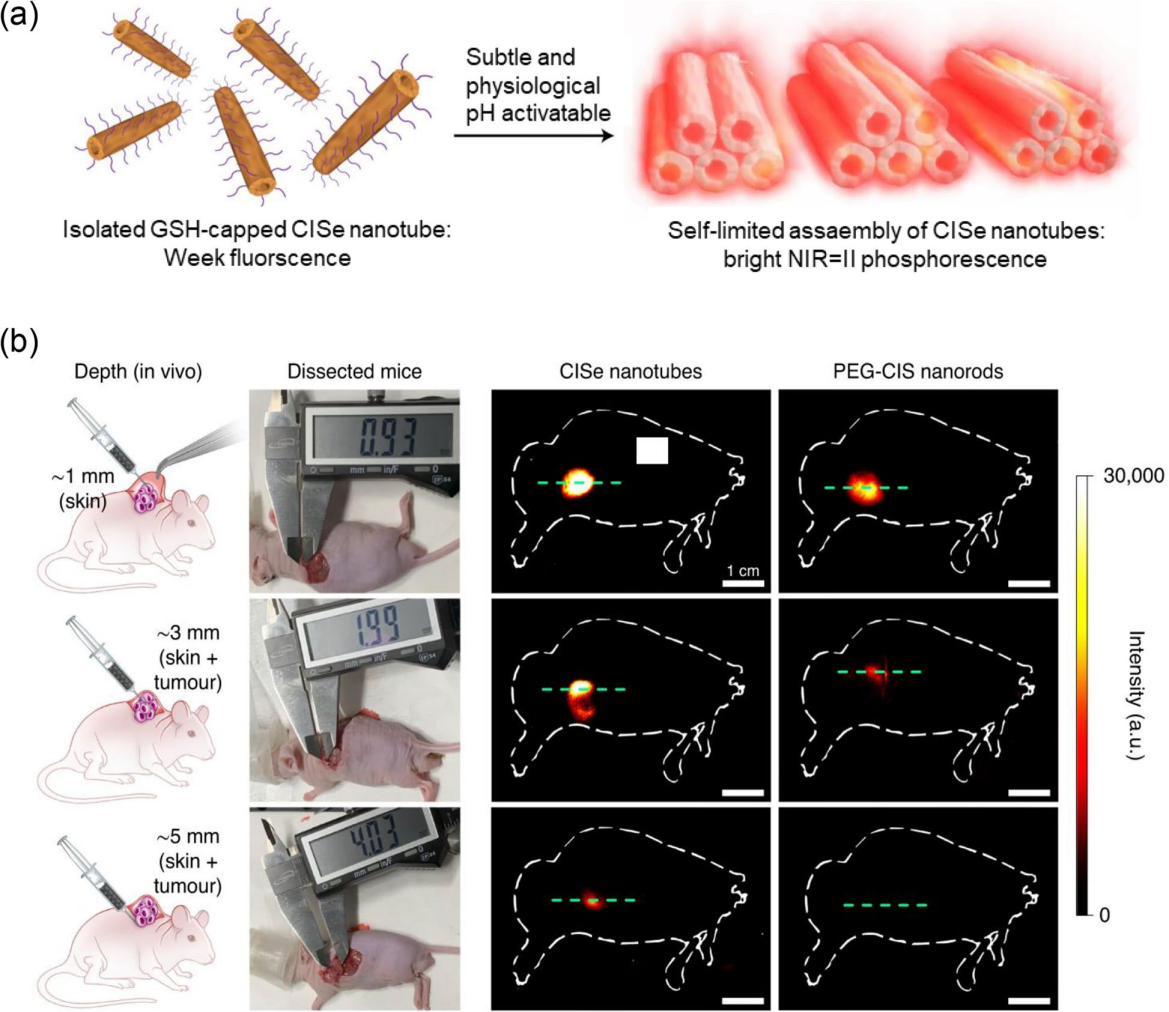
NIR-II phosphorescent probe for tumor imaging with superior penetration depth. **a** Schematic illustration of the assembled nanotubes. **b** Imaging signal attenuation test in tumor-bearing mice. Adapted with permission from Ref. [12]

Phosphorescence is a kind of photoluminescence with a longer emission lifetime (microseconds) than fluorescence (nanoseconds). The background signal caused by autofluorescence is a major problem in fluorescence imaging, which can be eliminated by time-resolved imaging using long-lived phosphorescence. As a part of these studies, Chang et al. introduced the NIR-II phosphorescent probe activated in a tumor acidic microenvironment [12] (Fig. 2a). They fabricated nanotubes with a diameter of 11.9 nm and a length of 170 nm composed of Cu, In, and Se, and capped them with glutathione (GSH). The nanotubes showed negligible fluorescence near pH 7, and when the pH dropped below 6, they formed clusters due to GSH, and the NIR-II emission intensity increased 5.8 × 10^3^-fold while switching from fluorescence to phosphorescence. The phosphorescence lifetime of the nanotubes was 336 μs, which was much longer than that of fluorescence (73 ns). Time-resolved NIR-II imaging was performed after intravenous injection of nanotubes at a dose of 20 mg/kg into 143B osteosarcoma tumor-bearing nude mice. In time-resolved NIR-II imaging, the fluorescence signal of the PEG-coated nanorods was filtered due to their short emission lifetime. On the other hand, the nanotubes with long emission lifetimes were able to image tumors without autofluorescence. Furthermore, to evaluate the advantage of NIR-II phosphorescence in penetration depth, in vitro imaging was performed while the sample was covered with 1% intralipid mimicking living tissue. The phosphorescent nanotube had a signal-to-noise ratio of about 15.6 in 5 mm thick intralipid, whereas the fluorescent nanorod was 1.9. To evaluate the penetration depth in vivo, the skin around the tumor was incised, the nanotubes were intratumorally injected at a depth of 5 mm, and then NIR-II phosphorescence imaging was performed (Fig. 2b). In the case of mice injected with nanotubes, a NIR-II signal was detected, but mice injected with PEG-coated nanorods showed negligible signals in the tumor region. These studies showed that the NIR-II region is more advantageous for optical imaging than visible or NIR-I region. However, there are not many probes matching NIR-II, so that more need to be developed for further applications.

### Bioluminescence

Light can be generated and emitted by living organisms such as fungi, bacterial strains, and marine organic microbes, and this phenomenon of naturally emitting light is known as bioluminescence. Bioluminescence was noticed by Robert Boyle in 1667 and used as an imaging tool after the mechanism was identified by other researchers [25]. The bioluminescence occurrence in the 400–600 nm wavelength range is caused by a reaction between the luciferin substrate and the luciferase enzyme. When luciferin is oxidized by its partner luciferase, a bright light is produced. Luciferase (FLuc) and D-luciferin derived from fireflies are representative examples of bioluminescent materials. There are several natural substrates used in bioluminescence, such as coelenterazine (CTZ) derived from sea creatures such as deep-sea shrimp, krill, bacteria or fungi. Furthermore, because the luciferase-based luminescence probe emits light rather than being excited by a light source, there is no background autofluorescence signal, allowing for high sensitivity in deep tissue imaging [25]. Recently, an engineered bioluminescence system that complements the limitations of existing materials has been developed, allowing detection of higher signals, and enabling bio-imaging in a multitude of settings such as imaging of tumors located in deeper tissues.

Yeh et al. suggested that a new bioluminescence pair of a modified coelenterazine analog and NanoLuc mutant would generate a red-shifted bioluminescence [13]. Most bioluminescence reporters emit at 400–600 nm, and their permittivity to deep tissues is low, making them unsuitable for deep tissue imaging in mammals. Even though the FLuc–D-luciferin pair with a wavelength of over 600 nm is the only one used for deep tissue imaging, efforts are being made to use a longer wavelength. Previous studies attempted to red-shift luciferin substrates by changing their chemical structure, but there is a limitation in that the reduced total intensity of emitted light eventually renders the spectral shift meaningless. The authors created a coelenterazine analog (DTZ, diphenyltetrazine) and NanoLuc mutants (TeLuc, Antares, Antares2) and obtained red-shift and high emitting signal effects. This raises the prospect of new bioluminescence reporters and bioluminescence engineering technology. DTZ, a CTZ analog, was modified at the CTZ structure’s C-8 position. TeLuc was established through the genetic recombination of NanoLuc, and Antares and Antare2 were created for BRET (bioluminescence resonance energy transfer)-based reporters by inserting the fluorescent proteins CyOFP1 into NanoLuc and teLuc, respectively. When the TeLuc–DTZ pair was evaluated in vitro, it was 46-nm red-shifted compared to the NanoLuc–furimazine pair, and the total intensity was improved by 2.6 times. When tested with 30 μM substrates and 100 pM proteins in the 650 nm region, the Antares–furimazine pair was 17 times brighter, and Antares2–DTZ pair was 65 times brighter than traditional FLuc–D-luciferin pair. In vivo evaluation with transfected Human Embryonic Kidney (HEK) 293 T cells demonstrated dramatically improved brightness and application potential in deep tissue. Following an intravenous injection of a specific amount of luciferase-expressing plasmid into BALB/c mice, the same concentration of luciferin substrates was injected intraperitoneally to analyze the emission photon. Antares–furimazine and Antares2–DTZ were 25 and 43 times brighter than FLuc–D-luciferin, respectively. The authors investigated the effectiveness of improved signal and red-shifted bioluminescence for deep-tissue imaging to overcome the limitations of traditional bioluminescence systems.

Nomura et al. developed a bioluminescence probe that emits light in response to biothiol-like cysteine [14]. To make CTZ generate light in the blue-shifted region, CTZ in this system was modified with an acryloyl group that is responsive to biothiols. In the resulting acryloyl methocy-CTZ-methoxy (AMCM), the acryloyl group was cleaved by biothiol, and light was emitted by the luciferase RLuc8.6–535. Luciferase was produced by transforming COS-7 cells to express a new RLuc protein, RLuc8.6–535, which increases the intracellular half-life of RLuc more than the traditional one. The imaging sensitivity of this system was increased by BRET, which is based on the genetic fusion of iRFP713 and RLuc8.6–535. The bioluminescence light from AMCM and RLuc8.6–535 can be shifted from a wavelength of 400 nm to a NIR wavelength through BRET with neighbor iRFP713. Because bioimaging in the NIR wavelength region is less affected by loss from surrounding tissues, highly intense signals can be obtained in vivo*,* and these are useful for deep tissue imaging. The strength of the bioluminescence signal increased when AMCM was treated with biothiol, cysteine and then incubated with RLuc8.6–535, but this change depended on the concentration of cysteine. For in vivo imaging via the BRET effect of this system, the transformed COS-7 cell that expresses iRFP713-RLuc8.6–535 was inoculated in 6-week-old female nude mice (BALB/c nu/nu). Then, AMCM was injected subcutaneously, and the bioluminescence intensity was measured in a NIR region. The mice expressing BRET proteins showed strong intensity compared to mice with tumor cells without BRET protein. The signal difference of nearly 15 times or more was observed in the prone position deep tissue imaging results. This result demonstrated the high applicability of this system based on BRET in deep tissue imaging.

**Fig. 3:**
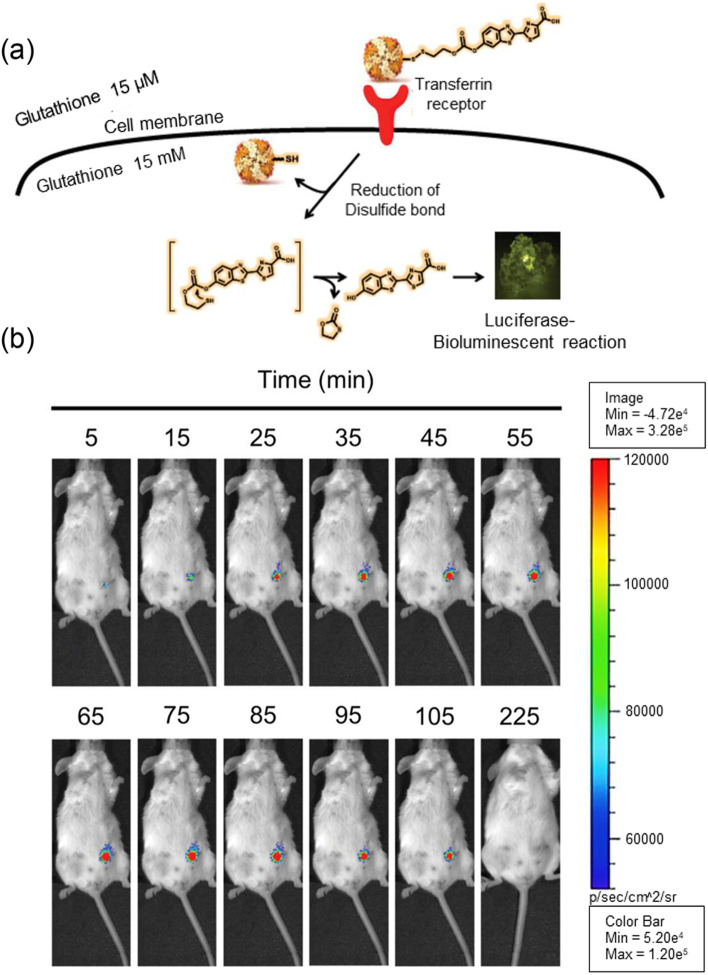
Bioluminescent imaging probe for tracking of the injected nanoparticles in cancer tissue. **a** Schematic illustration of the ferritin nanocage conjugated with luciferin. **b** Time-dependent bioluminescence signals in mice after intravenous injection of the probe. Adapted with permission from Ref. [15]

Recently, Bellini et al. showed that bioluminescence imaging could be used for developing a sensitive tool for analyzing the delivery of nanoparticles into tumor cells [15]. The authors used H-ferritin (HFn) nanocages and conjugated luciferin to them via a cleavable disulfide linkage (Fig. 3a). HFn binds the transferrin receptor 1 (TfR1) that is overexpressed in many cancers. After binding and cellular uptake of the nanocage into tumor cells, free luciferin was released and reacted with luciferase in tumor cells. The self-immolative spacer was used to trigger intramolecular reaction after disulfide cleavage and released luciferin for an efficient bioluminescence reaction. The nanocage itself could not generate bioluminescence with luciferase, but the light intensity increased more than 400 times in the reductive condition with dithiothreitol treatment. After intravenous injection of the nanocage into mice bearing luciferase-expressing 4 T1 breast cancer cells, intense bioluminescence signals were observed in the tumor area up to 105 minutes (Fig. 3b). There were no signals in other sites, illustrating the specificity of the nanocage and the advantage of bioluminescence with negligible background signals. This demonstrated that the proposed bioluminescence system could enable precise real-time in vivo imaging to observe the uptake of nanoparticles into tumor cells after injection. These recent studies proved the usefulness of bioluminescence for in vivo imaging as well as in vitro assays.

### Chemiluminescence

Chemiluminescence is a useful tool in deep tissue imaging. Rather than being excited by a light source, chemiluminescence emits light through a chemical reaction with reactive nitrogen species such as nitric oxide (NO), substances related to life activities in vivo like hydrogen peroxide (H_2_O_2_), and peroxynitrite (ONOO^−^), and azanone (nitroxyl, HNO), reactive sulfur species like hydrogen sulfide (H_2_S) and polysulfides (RSnH/RSnR), reactive carbon species like formaldehyde (CH_2_O) and carbon monoxide (CO), and oxygen (O_2_)/hypoxia. Because it is not an external light-based imaging method, there is no concern about autofluorescence in the background, so it can provide highly sensitive deep tissue imaging. Chemiluminescence probes react by their own unique mechanisms, and new derivatives for emitting light in response to a specific reactant have been developed. 1,2-dioxetane, developed by Shabat and colleagues, is a representative chemiluminescence probe, and its derivatives such as AF1, 5-Fluoroisatin, and SF7-AM also have been developed [26]. Each derivative reacts and generates intense emission light with azanone (HNO), peroxynitrite (ONOO^−^), and hydrogen sulfide (H_2_S), respectively, which are produced by physiological activity. Furthermore, in vivo imaging performance evaluations of 1,2-dioxetane derivatives Galacton Plus (β-d-galacto pyranoside trigger), CHS-3, and HyCL probes confirmed the high-sensitivity imaging results. Galacton Plus emits light via β-galactosidase and was commercialized by Thermo-Fisher Scientific. A genetically engineered Lac Z tumor that enzymatically overexpresses β-galactosidase showed 10 times the emission intensity of wild-type mice in xenografted mice [27]. CHS-3 emitted light via H_2_S-mediated azide reduction and at a high intensity in the mouse model induced by A549 lung epithelial cancer cells [28]. HyCL probes were applied to the characteristics of the hypoxia environment of tumor tissue, and imaging was used to diagnose tumor treatment and growth. Intratumoral injection of HyCL-2 into xenograft model mice injected with human lung tumors resulted in a higher signal in a hypoxia environment with a 21% oxygen volume ratio [29]. These studies showed that 1,2-dioxetane and its derivatives are highly versatile chemiluminescence probes with diverse and highly sensitive in vivo imaging, and research is ongoing for advanced potential clinical monitoring and analytes.

Among probes, Scott et al. developed the first chemiluminescent probe for in vivo imaging of NK cell activity [16]. All living things have a perfect autoimmune system as a defense against external threats. Cancer is primarily caused by genetic mutations. To treat these, living organisms mature lymphocytes that express many active substances in the immune system and induce cell apoptosis. Natural killer (NK) cells are a type of cancer immunotherapy that uses adoptive cell transfer to remove mature lymphoid cells from tumors like AML and ovarian cancer. NK cell activity in tumor patients can be used as an excellent imaging probe for diagnosis and treatment. When NK cells recognize a tumor cell, they release cytolytic granzymes that cause tumor cell apoptosis. Using these features, the author created a phenoxydioxetane probe that specifically binds granzyme B and emits light and demonstrated its potential as a chemiluminescent probe in vitro and in vivo. The probe consists of a cleavable construct by granzyme B and a light-emitting phenoxydioxetane. The probe mediated granzyme B concentration- and time-dependent chemiluminescence signal intensity in co-cultured MDA-MB-231 (human breast adenocarcinoma cell line) and human NK-92 cells, which were similar to those observed for in vivo conditions. The probe generated greater chemiluminescence in the co-culture environment than in the single culture environment of each cell. The author also examined the probe’s efficacy in a xenografted mouse model. After injecting MDA-MB-231 cells subcutaneously into immunocompromised mice, NK-92 cells were injected into the tumors, and the efficacy of the new probe was also assessed after intratumoral injection. As expected, the chemiluminescence probe was activated where NK-92 cells were injected. Because chemiluminescence was detected based on the activation of NK cells, it was free from background autofluorescence and the limits of existing optical imaging techniques requiring light sources. Thus, it showed sufficient potential in deep tissue imaging.

**Fig. 4:**
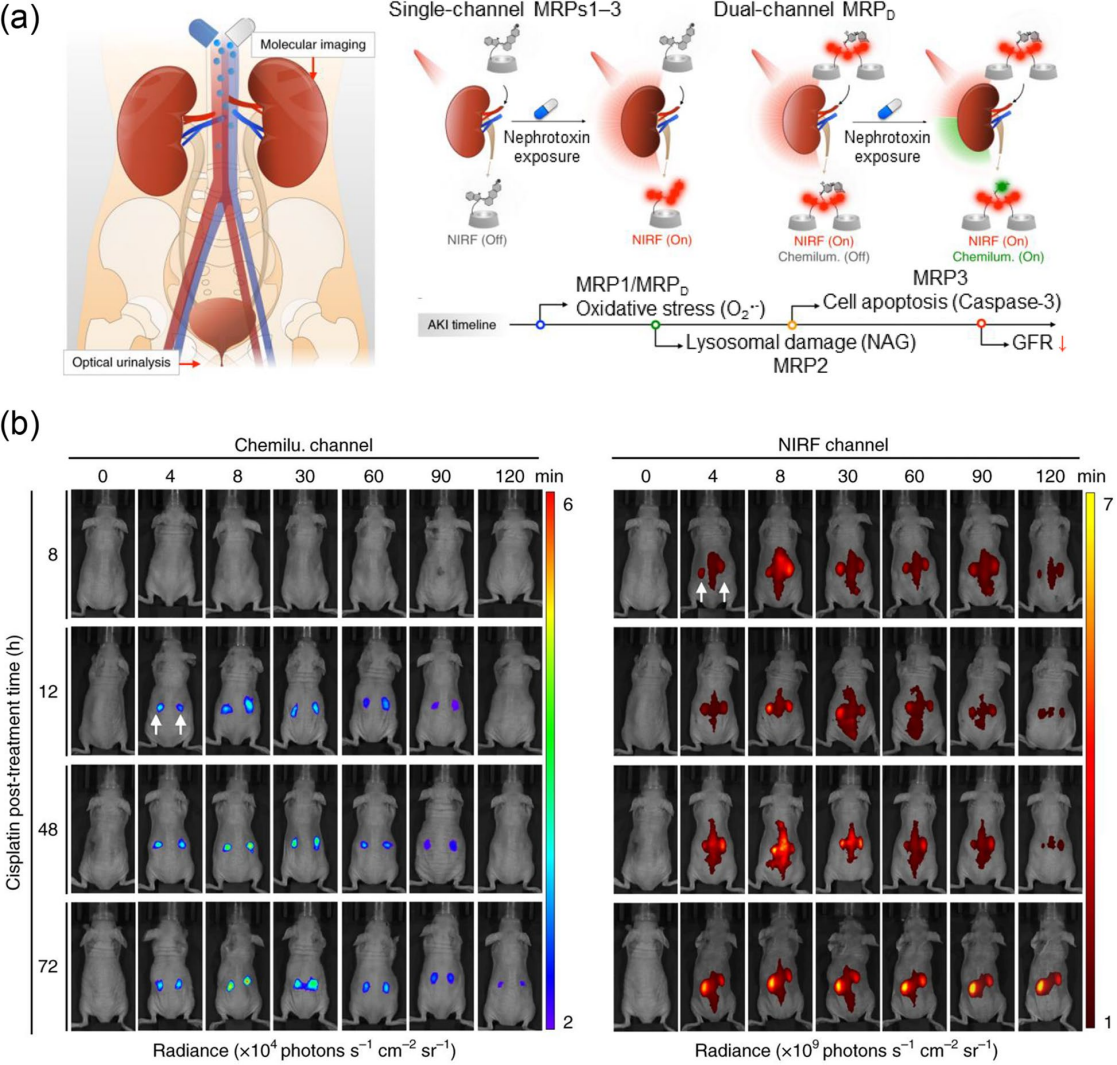
Chemiluminescent imaging probe for diagnosis of acute kidney injury. **a** Schematic illustration of the chemiluminescence probe with three components. **b** Chemiluminescence/NIRF dual imaging of acute kidney injury in mice. Adapted with permission from Ref. [17]

Pu’s group introduced a chemiluminescent imaging probe that can detect drug-induced acute kidney injury (AKI) quickly [17]. FDA-approved representative biomarkers N-acetyl-beta-D-glycosaminidases (NAG), inflammatory mediators (trefoil factor-3 (TFF3), inflammatory mediators (trefoil factor-3 (TFF3)), glomerular filtration markers (Cyst C) and β2-microglobulin (β2-Mic) are used to diagnose kidney damage. However, there is a limitation in that these can be detected after the disease has advanced significantly, making adequate treatment unavailable to the patient. As a result, researchers created novel in vivo imaging probes that react with other AKI-related molecules as well as existing biomarkers to aid in rapid diagnosis. The developed MRP_D_ probes are chemiluminescence probes that emit light at 520 nm by reacting with superior oxygen (O_2_^−^) in the absence of excitation light, and an a NIR probe that emits light always from the NIR fluorophore without reaction with superior oxygen (Fig. 4). This technique is a dual-channel probe (i.e., a chemiluminescence and NIRF probe) because it was synthesized from three small molecules: Cy7 (a NIRF signal structure), CySCL (a chemiluminescence signal structure), and (2-hydroxypropyl)-cyclodextrin (HPCD) (a part of renal clearance). Cisplatin was injected into mice for in vivo AKI modeling. When the probe was injected intravenously at 8, 12, 48, and 72 hours, a chemiluminescence signal was observed starting 12 hours after injection. This is 60 hours faster than the point at which the glomerular filtration rate (GFR) function decreases to less than 50%. Dual imaging with NIRF showed high accumulation of the probe in kidneys, and negligible background signal in other tissues showed the advantage of chemiluminescence. This novel AKI diagnostic-associated probe provided a strong detection signal in kidneys through deep tissue in a short period of time and resulted in rapid renal clearance after imaging. This system has the potential to be applied to numerous disorders associated with biomarkers besides AKI diagnosis.

Oxidative damage to nucleotides, proteins, and lipids by endogenous singlet oxygen (^1^O_2_) is related to cell death and is an important step in pathological processes and immune response. Therefore, many researchers have attempted to develop non-invasive imaging techniques to monitor ^1^O_2_. In 2019, Zhang et al. suggested a chemiluminescence-based nanosensor for real-time ^1^O_2_ imaging [18]. For improved sensitivity and retention time of imaging, the authors used aggregation-induced emission (AIE) material containing an ultrahigh concentration of tetraphenylethylene-phthalhydrazid (TPE-PH), a chemiluminescence dye responsive to ^1^O_2_. The intramolecular energy transfer between TPE-PH molecules and their high quantum yield resulted in intense signals for a long time. The resulting nanoparticle TPE-PH (NTPE-PH) is responsive to only ^1^O_2_ among various reactive oxygen species containing H_2_O_2_, ClO^−^, OH^.^, and O^.^_2_^−^. The detection limit of NTPE-PH was calculated to be 4.6 × 10^− 9^ M ^1^O_2_. When NTPE-PH was encapsulated with Pluronic F127 to prevent aggregation, its chemiluminescence performance was maintained at 93% after 4 weeks of storage at room temperature. After intravenous injection of NTPE-PH into rat models, the maximum target/nontarget ratios of chemiluminescence were about 35 and 5 in arthritis induced by lipopolysaccharide and pristine samples, respectively. The intense signal could be observed in tens of minutes, which was enough time for monitoring. The promising results in animal imaging showed real-time monitoring of ^1^O_2_ could be enabled based on chemiluminescence without external light irradiation. These studies demonstrated the unique property of chemiluminescence that there can be many chemical reactions targeting various molecules, which indicates its potential in deep tissue imaging.

### Afterglow (persistent luminescence) imaging

Persistent luminescence (PersL) is the emission of afterglow luminescence after termination of excitation light. Persistent luminescence is caused by excited electrons in a trap, an energy state produced by intrinsic defects or doping. When electrons in emitters are excited, electrons are captured in traps. Once the excitation is stopped, electrons returned to the ground state, emitting persistent luminescence. Persistent luminescence is independent from real-time excitation, which leads to less auto-fluorescence. In addition, various excitation sources of PersL enable deep penetration. In particular, PersL of NIR has low absorption and scattering, which reduces light attenuation. Those advantages of PersL result in highly sensitive imaging and enhanced deep tissue imaging. Recently, nanoparticles emitting PersL were studied for effective deep tissue imaging. Persistent luminescence nanoparticles (PLNPs) both emit PersL and serve as carriers loading various imaging modalities and drugs [30]. Sizes and morphologies of PLNPs were controlled exquisitely through diverse synthesis methods such as sol-gel, template or hydrothermal methods. In addition, biological ligands can be attached onto PLNPs by surface modification, which reinforces bioactivity and biocompatibility. Doping and modification of PLNPs with other imaging agents enable multimodal imaging for more sensitive and accurate disease diagnosis. Mesoporous and hollow structures of PLNPs can be used to make therapeutic agents loaded in nanoparticles, leading to effective theranostics. Although there are remaining limitations of PLNPs, including further precise control of characteristics and delivery of NP, and biosafety issues, PLNPs showed notable effects in excitation-free imaging, image-guided surgery, and theranostics by combining them with several therapies. In particular, PLNPs with NIR PersL showed high signal-to-noise ratio (SNR) and enhanced PersL time for efficient deep tissue imaging in several studies.

Li et al. suggested 5 nm ZnSn_2_O_4_:Cr,Eu (ZSO) NIR emitting persistent luminescence nanoparticles (NPLNPs) with PersL emission at 800 nm [19]. Compared to sol-gel or solid-state methods, ZSO NPLNPs were synthesized directly in an aqueous phase by the hydrothermal method, which led to controlled sizes around 5 nm with a narrow distribution. Abundant hydroxyl groups on ZSO NPLNPs could be used for facile functionalization with biomolecules such as folic acid (FA), a tumor targeting reagent. Cr^3+^ and Eu^3+^ ions in ZSO PLNPs served as emitting and trap centers and brought out NIR PersL at 800 nm more than 30 min after removal of the UV lamp. In addition, this NIR emission was re-stimulated by NIR excitation at 650 and 808 nm. Enhanced NIR PersL of ZSO NPLNPs effectively penetrated 2.5 cm of pork tissue with a high SNR of 7.39 and showed successful in vivo penetration for more than 15 min with a significant SNR of 25.5 in normal mice. Furthermore, ZSO functionalized with folic acid (ZSO-FA) NPLNPs revealed notable targeting ability to folic acid-abundant tumors in vitro and in vivo. ZSO-FA were internalized into MCF7 6.33 times better than ZSO in vitro, and 29.08% of ZSO-FA NPLNPs were accumulated at tumor sites of MCF7 bearing mice after i.v. administration. NIR afterglow luminescence of ZSO-FA NPLNPs in tumors showed a remarkable SNR of 13.3, which remained for than 10 min, and was re-excited effectively with 650 nm and 808 nm light, enabling long-term and deep tissue imaging for accurate diagnosis.

**Fig. 5:**
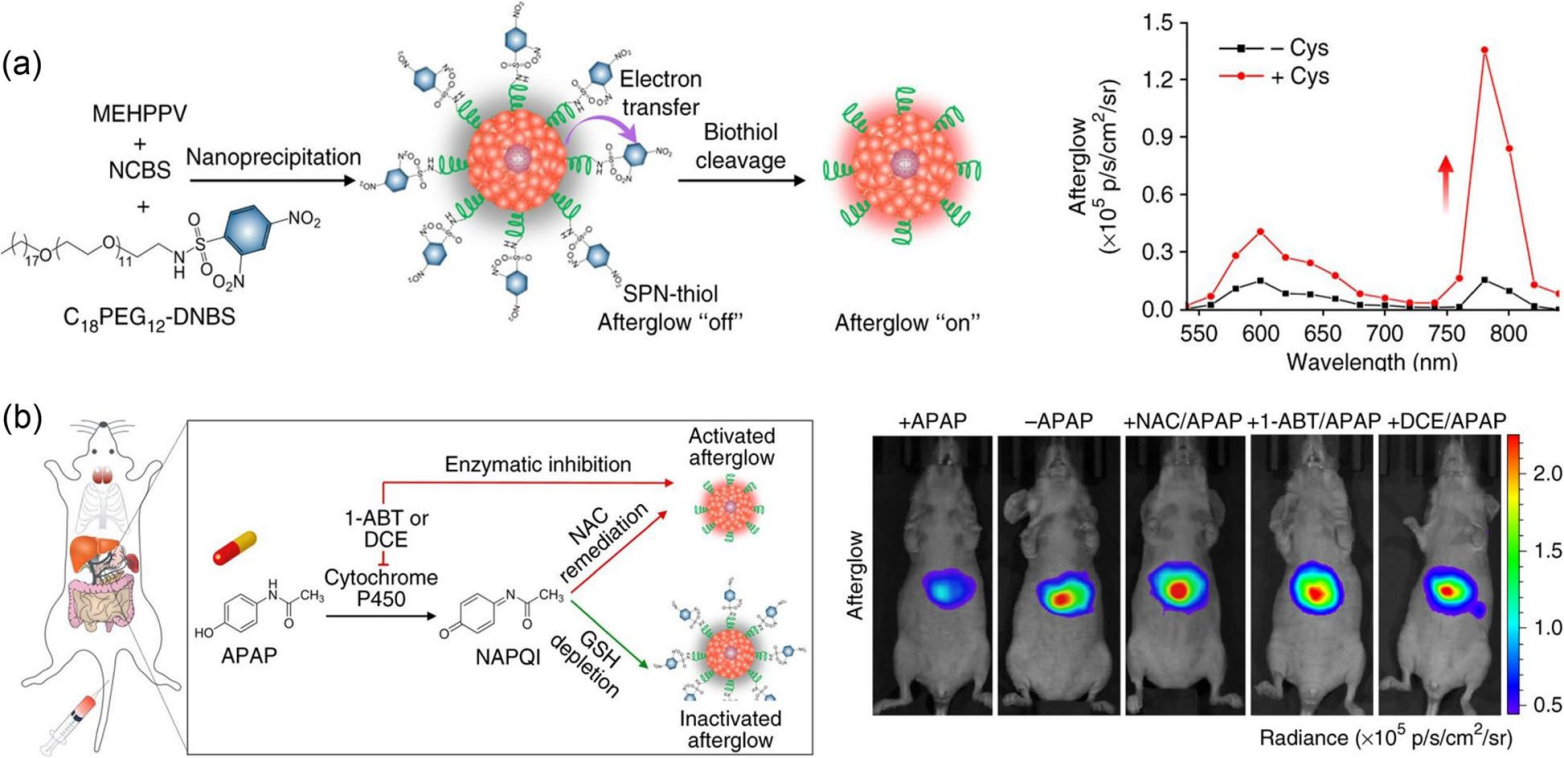
Afterglow imaging probe for in vivo imaging of drug-induced hepatotoxicity. **a** Schematic illustration of the persistent luminescence nanoparticle and luminescence signal data. **b** Persistent luminescence imaging in liver of the mice treated with acetaminophen. Adapted with permission from Ref. [20]

There were several attempts to construct PLNPs using organic materials with efficient afterglow luminescence. Pu’s group reported semiconducting polymer nanoparticles (SPNs) with red-shifted and enhanced NIR afterglow luminescence at 780 nm [20]. SPNs were prepared by nanoprecipitation of diverse semiconducting polymers(SPs) and PEG-*b*-PPG-*b*-PEG triblock copolymers (Fig. 5a). Among several SPs, phenylenevinylene(PPV)-based SPNs showed afterglow luminescence, since singlet oxygen generated by irradiation oxidized vinylene bonds of PPV, leading to unstable PPV-dioxetane intermediate emitting afterglow luminescence. In addition, for amplification and red-shift of afterglow luminescence, they doped a ^1^O_2_ sensitizer, silicon 2,3-naphthalocyanine bis(trihexylsilyloxide) (NCBS), into SPN-MEHPPV with the most intensive afterglow luminescence among PPV-based SPNs. SPN-NCBS showed amplified NIR afterglow emission at 780 nm. An 11-fold increase in afterglow luminescence was observed after pre-irradiation at 808 versus 514 nm. Based on these results, SPN-NCBS revealed a magnificent signal-to-background ratio (SBR) of 291, which is 67 times larger than that of NIR fluorescence in the detection of NIR signals through 1.5 cm thick chicken tissue. In addition, SPN-NCBS was effectively accumulated into lymph nodes and tumors in vivo and showed efficient afterglow luminescence only after 2 hours with a significant SBR of 149.7 in the tumor, 23.3-fold higher than that of NIR fluorescence. The surface of SPN-NCBS was also modified with amphiphilic oligomer with quencher (C_18_PEG_12_-DNBS), resulting in selective cleavage in the presence of biothiols including cysteine and glutathione. Based on a reduction of afterglow luminescence of SPN-thiol with deceasing antioxidants, SPN-thiol effectively detected drug-induced hepatotoxicity involving the consumption of antioxidants against oxidative stress (Fig. 5c). These results demonstrated promising organic PLNPs with remarkable NIR afterglow luminescence for effective and selective in vivo imaging.

Further investigation to identify more biocompatible nanoparticles emitting afterglow luminescence resulted in the development of organic afterglow luminescent nanoparticles. Pu’s group also proposed afterglow luminescent nanoparticles (ALNPs) using traditional fluorescent agents through a cascade photoreaction, which stores energy in chemical defects and results in delayed emission after termination of excitation [21] . In the cascade photoreaction, a photosensitizer served as an afterglow initiator to produce ^1^O_2_, leading to the generation of an unstable chemiluminescent intermediate (1,2-dioxetane) after a reaction between ^1^O_2_ and afterglow substrate (^1^O_2_ reactive molecule). Then, unstable intermediate transfer energy to fluorescent substances of an afterglow was relayed through chemically initiated electron exchange luminescence (CIEEL). The wavelength of afterglow luminescence is defined according to the existence of secondary energy transfer (SET) between the afterglow relay and initiator. Screening of three components consisting of ALNPs was conducted to predict optimized compositions via mathematical predictions. Afterglow luminescence from simulations and experiments were similar, revealing that all three components affected afterglow intensity. Among diverse compositions, optimized ALNPs consisted of silicon 2,3-naphthalocyanine bis(trihexylsilyloxide) (NCBS) as an afterglow initiator, (*N,N*-dimethyl-4-(3-phenyl-5,6-dihydro-1,4-dioxin-2-yl)aniline) (DO) as an afterglow substrate, and a semiconducting polymer of poly[(9,9′-dioctyl-2,7-divinylenefluorenylene)-alt-(9,10-anthracene)] (PFVA) as an afterglow relay (PFVA-N-DO). PFVA-N-DO showed NIR afterglow emission at 780 nm, leading to effective tissue penetration with a SBR of 248 at a thickness of 2 cm and an SBR of 26 at a thickness of 5 cm. Additionally, the afterglow intensity of PFVA-N-DO sharply increased only 1 h after intravenous administration with a SBR of 2922, which enables earlier and more sensitive tumor detection compared to NIR fluorescence. PFVA-N-DO showed effective biodegradation in the presence of myeloperoxidase (MPO) in phagocyte, and an undetectable level was observed 33 days after administration in vivo. These results indicate the notable possibility of biocompatible ALNPs for further improved applications in effective deep tissue and in vivo imaging. These studies suggest the encouraging possibility of PLNPs for deep tissue imaging and diagnosis, but time-dependent changes in signal intensity need to be carefully considered in every case of afterglow imaging.

## Technical improvement for deep-tissue imaging based on adaptive optics

**Fig. 6:**
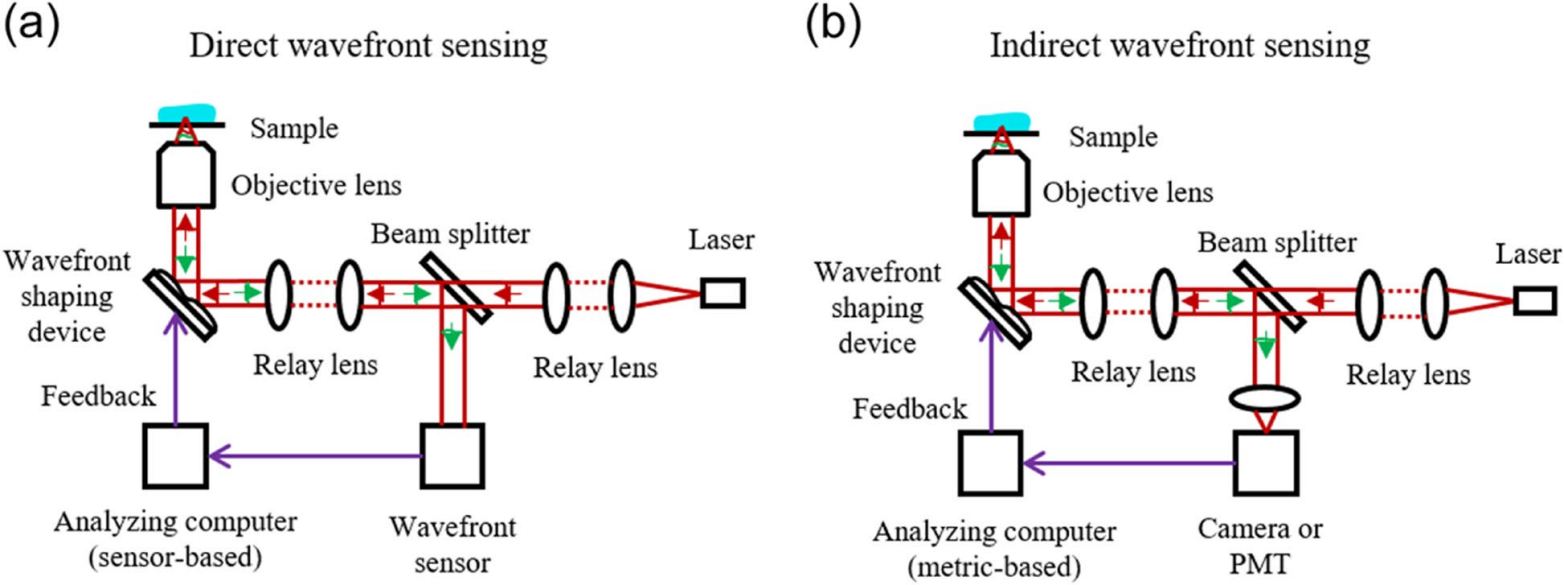
Diagrams of AO techniques. **a** Direct wavefront sensing method using a wavefront sensor to directly measure the wavefront. **b** Indirect wavefront sensing method to optimize the image-based metric

Deep-tissue imaging based on adaptive optics (AO) has been intensively studied for several decades. Methodologies for measurement of optical aberration can be classified into (i) direct wavefront sensing with a wavefront sensor and (ii) indirect wavefront sensing with an image-based metric [4, 31, 32] (Table 2). The principle of direct and indirect wavefront sensing for AO is shown in Fig. 6. The wavefront sensor (Fig. 6a) measures the optical aberrations in the direct sensing method. Otherwise, the wavefront sensor is not installed in the indirect sensing method (Fig. 6b), which is also referred to as sensorless AO.

**Table 2:** Exemplary imaging applications based on optical techniques

Optical techniques	Advantages/Obstacles	Exemplary imaging applications	Ref.
Direct wavefront sensing	High sensing speed / Require wavefront sensors, Need fluorescent labeling, weak at scattering	Oligodendrocytes and neuronal nuclei in a zebrafish brain in vivo	[33]
		Neurons in a Thy1-YFPH mouse brain in vivo	[34]
		mRuby2-labeled layer 5 pyramidal neurons of vS1 mouse brain cortex in vivo	[35]
		A live human stem cell-derived organoid	[36]
		The eye of a zebrafish embryo 24 hpf.	[36]
		Cortical neurons of a Thy1-GFP mouse brain in vivo	[37]
		*C. elegans* in vivo expressed by the adherens junction marker ajm-1::GFP	[38]
Indirect wavefront sensing	Better suited to opaque tissues than direct wavefront sensing, Require no wavefront sensors / Slow sensing speed due to hardware feedback, Deal with low-order aberrations modes, Need fluorescent labeling	The visual cortex and Hippocampus of mouse brain in vivo	[39]
		Synaptic structures in the deep cortical region of a Thy1-GFP mouse brain	[40]
		High and basal dendritic spines of a mouse V1 neuron in vivo	[41]
		GFP expressed microtubule of Drosophila larval macrophage	[42]
		GFP-expressed pyramidal neuron in a living mouse brain	[43]
		Single microglia cell from hippocampus tissue of a mouse brain	[44]
Coherence-gating	Label-free, High sensing speed / Mixed phase retardations of input and output paths, Deal with low-order aberration modes	Olfactory bulb in transgenic zebrafish larvae	[45]
Time-gated reflection matrix	Label-free, Better suited to opaque tissues, Numerical post-processing, Seperation of input and output aberrations, High- order aberration correction / Matrix acqusition time depending on scattering	Hyphae of Aspergillus cells in a rabbit’s cornea	[46]
		Nerve system and hindbrain of a 10-dpf larval zebrafish in vivo	[47]
		In vivo through-skull mouse brain imaging	[48]
		ex vivo through-skull neuronal dendrites in the brain of a Thy1-EGFP line M mouse	[48]

In terms of the imaging speed, it takes several or tens of milliseconds to measure and correct the wavefront in the direct sensing method. However, the indirect sensing methods are slower than the direct sensing methods because of the time required for calculation of the metric during the optimization process. The indirect sensing method typically requires a few seconds to tens of seconds. In terms of the optical property of the sample, direct sensing works well for cultured cells or transparent samples [49–60]. Whereas, indirect sensing can measure and correct the wavefront even on relatively opaque tissue samples in comparison with the direct sensing method [61–75]. In addition, the indirect sensing method is more suitable when the system aberrations of the microscope and the sample-induced aberrations are mostly static. The indirect measurement is easy to implement in hardware because it does not require an additional wavefront sensor.

### Direct wavefront sensing for AO fluorescence imaging

In direct wavefront sensing, the wavefront sensor (e.g., the Shack-Hartmann sensor) is used to determine the optical aberrations. The procedures of measurement and correction of the optical aberrations in direct wavefront sensing are as follows. At first, the aberration-free wavefront is measured with an aberration-free sample in each lens array of the sensor. Then, the displacements of the foci by the optical aberrations are measured in the sample. The local slope of wavefront segment is calculated, and the value is applied to the wavefront shaping device such as a deformable mirror.

The direct sensing method is advantageous because it can be used to rapidly measure the optical aberrations in tens of milliseconds. However, a guide star that emits a sufficiently strong signal is needed close to the region of interest to precisely measure and correct the optical aberrations. For example, fluorescent beads injected into the biological sample can be used as a guide star. A two-photon or three-photon microscope, however, can illuminate a focal volume and use this localized signal as a guide star. These pinhole-free microscopes use a laser source of the infrared wavelength and can be used to improve the imaging depth with reduced scattering compared to the visible wavelength. The method works well only in transparent or weak scattering samples, i.e., cultured cells or transparent tissues, since the signal of the guide star gets worse along the depth of the sample.

**Fig. 7:**
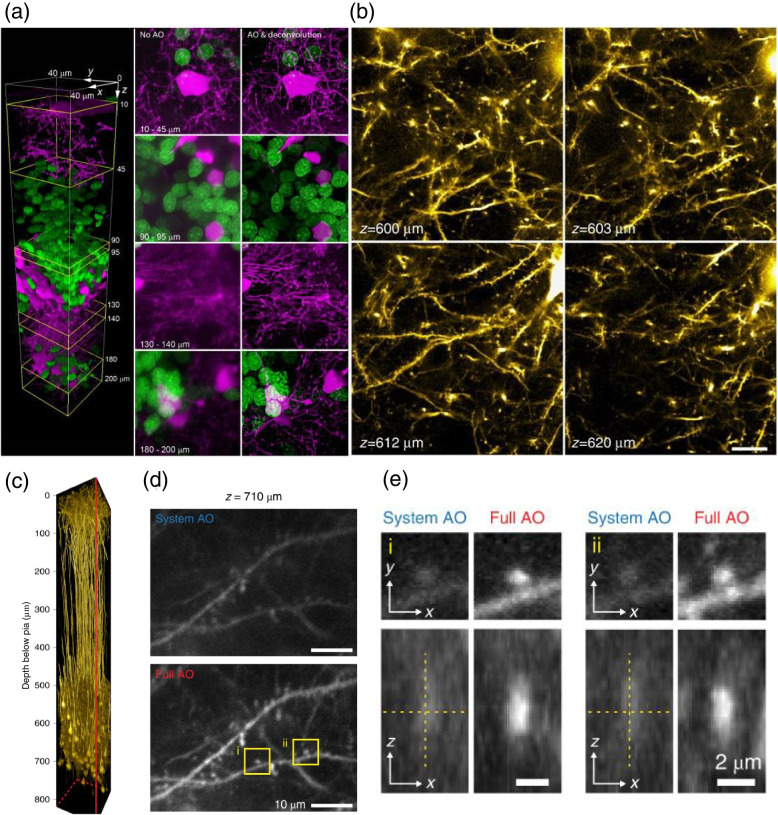
Direct wavefront sensing with focus scanning microscopy. **a** The three-dimensional volumetric images of oligodendrocytes (magenta) and neuronal nuclei (green) in a zebrafish brain in vivo. Adapted with permission from Ref. [33]. **b** Thy1-YFPH neurons at 600–620 μm in a mouse brain in vivo. Adapted with permission from Ref. [34]. **c-e** mRuby2-labeled layer 5 pyramidal neurons in a 150 × 150 × 810 μm^3^ of vS1 mouse brain cortex in vivo. Adapted with permission from Ref. [35]. Scale bar in b, 20 μm. Scale bar in d, 10 μm. Scale bar in e, 2 μm

The direct sensing method is easily applicable for focus scanning fluorescence microscopy (Fig. 7). The two-color confocal volumetric images of a living zebrafish brain were acquired by a two-photon guide star (Fig. 7a) [33]. The left panel is the three-dimensional volumetric image of oligodendrocytes (magenta) and neuronal nuclei (green). The center is the maximum intensity projection (MIP) before aberration correction, and the right is the MIP after correction through the depth of 200 μm [33]. The direct wavefront sensing is implemented in the high numerical aperture system to measure and correct the complex sample-induced aberration by a laser-induced guide star with the high-speed aberration correction at a 14-ms update rate.

Direct sensing with a two-photon guide star is also suitable for the mouse brain imaging since the scattering in the biological sample is reduced in the near-infrared wavelength compared to the visible wavelength. The images of the mouse brain cortex in vivo about the depth of 600 μm were measured by the direct sensing with the two-photon guide star (Fig. 7b) [34]. The fluorescent images of neurons with the two-photon excitation were acquired in a Thy1-YFPH mouse at 600–620 μm after the correction with direct wavefront sensing. The field-of-view of the single plane was 120 × 120 μm.

The Cy5.5-conjugated dextran can be exploited for the two-photon guide star in the direct wavefront sensing [35]. This fluorescent conjugate material was supplied to the microvascular structure of a living mouse brain. The image in Fig. 7c was acquired by the mRuby2-labeled layer 5 pyramidal neurons in a 150 × 150 × 810 μm^3^ of vS1 cortex in vivo. The AAV-Flex-mRuby2 was injected into layer 5 of Rbp4-Cre KL100 mice. The excitation wavelength of the two-photon laser was 1.07 μm. Aberrations were corrected at every depth of 50 μm. The image in Fig. 7d is the dendritic spines 710 μm below the pia in Fig. 7c with system-aberration correction (top) and full-aberration correction (bottom). Figure 7e shows the zoomed-in views of postsynaptic spines in the boxed regions in Fig. 7d. Unlike the method of fluorescent dye injection, the cy5.5-dextran injection has the advantage of being continuously supplied to the microvessels of the mouse brain. Thus, it acquired the glutamate release from thalamocortical axons and calcium transients in spines of basal dendrites.

**Fig. 8:**
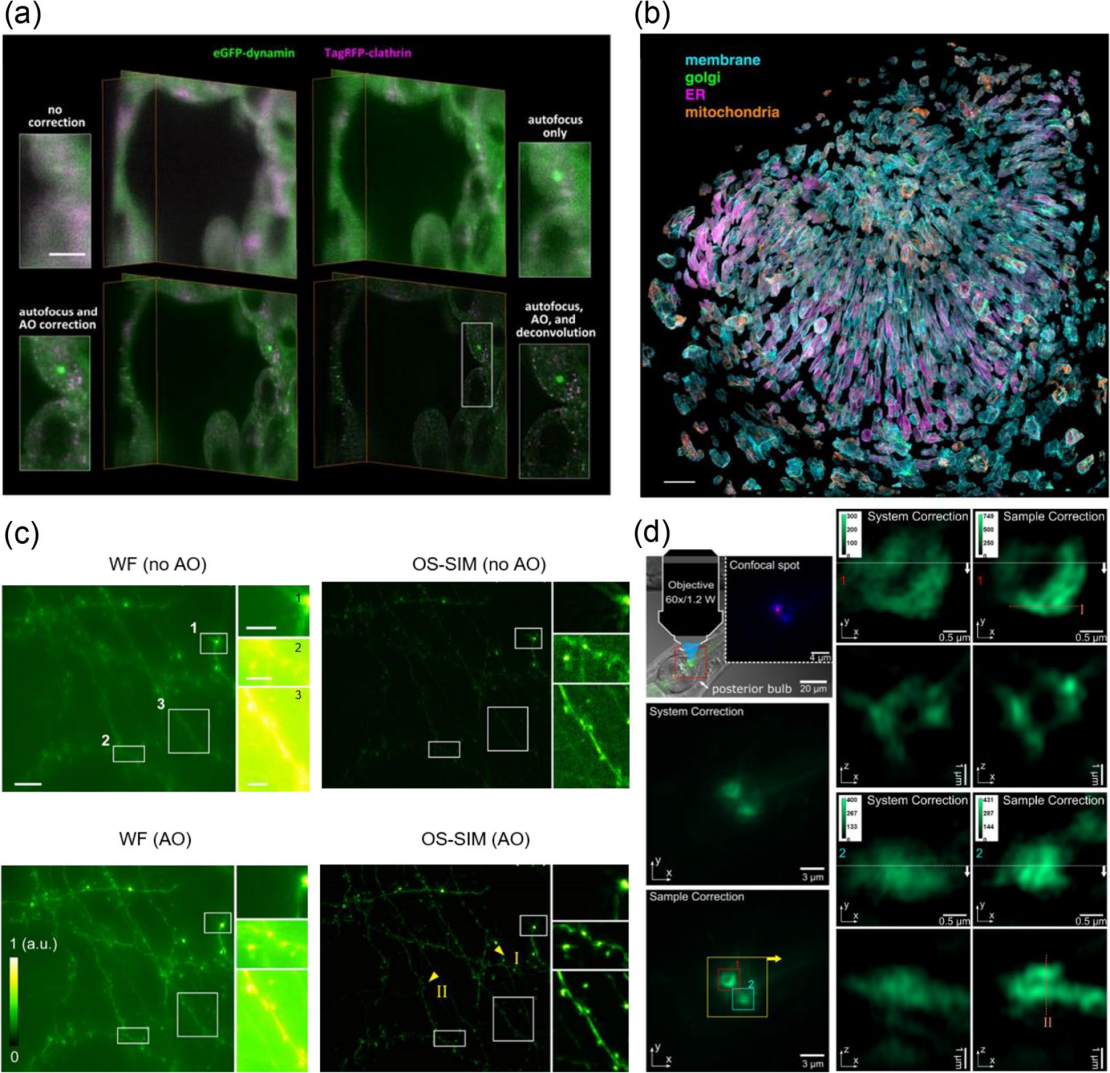
Direct wavefront sensing with LLSM and SIM. **a** A live human stem cell-derived organoid with four steps of corrections. Adapted with permission from Ref. [36]. **b** The eye of a zebrafish embryo 24 hpf. The separated cells are indicated in color. Adapted with permission from Ref. [36]. **c** Cortical neurons of a Thy1-GFP mouse brain in vivo. Adapted with permission from Ref. [37]. **d** *C. elegans* in vivo images expressed by the adherens junction marker ajm-1::GFP. Adapted with permission from Ref. [38]. Scale bar in a, 5 μm. Scale bar in b, 30 μm. Scale bar in c, 10 μm; inset, 3 μm

The lattice light-sheet microscopy (LLSM) can be combined with the method of direct wavefront sensing. LLSM combined with adaptive optics enabled to acquire three-dimensional images by moving the thin light sheet rapidly in the volume of interest. The region to be observed can be illuminated only by a thin light sheet, thereby avoiding photobleaching outside the volume. In addition, the instrument for direct wavefront sensing measured the sample-induced aberration and corrected it with fluorescent guide star in the volume. As an appropriate application, imaging a live human stem cell-derived organoid are shown in Fig. 8a [36]. The organoids are differentiated from human stem cells, gene-edited to express red fluorescent protein (tagRFP)–clathrin and enhanced green fluorescent protein (EGFP)–dynamin in endocytic pits. Significant wave distortion induced by the extracellular matrix of organoids requires precise aberration correction, while high-speed imaging is needed to measure the rapid dynamics of clathrin-coated pits. Hence, the property of the sample is suitable to evaluate the imaging performance of AO LLSM. Four steps of correction were identified to assess the imaging performance. The upper left panel shows the result without adaptive optics or focus correction. The clathrin-coated pits are not visible, and the boundary of the cell is not clear. The upper right panel shows the larger patches of clathrin and dynamin with autofocus alone. The lower left panel shows an image to identify individual clathrin-coated pits with an aberration correction for excitation and detection paths. From the diffraction-limited image, the data is enhanced by the deconvolution algorithm with the system-corrected point spread function. The lower right panel shows the dynamin and clathrin puncta clearly above the cytosolic background.

Observing the development of organism is another suitable application, however corrective updates of wave distortions are required during the development since the shape, position, or refractive index of the embryo changes. In general, the time scale of the developmental variation is longer than the detection time required to obtain large-area images using the AO LLSM. Volumetric imaging with corrective updates was applied to observe the development of zebrafish’s eyes (Fig. 8b) [36]. The cell shape and organ distribution were acquired during development of zebrafish’s eyes by correcting the curvature, refractive index, and aberration changes of the zebrafish using the AO LLSM. The images of the eye of the 24–27 hpf embryo were acquired over 4 × 4 × 3 tiles at 6-minute intervals and segmented, isolated each cell was visualized.

The structured illumination microscopy (SIM) can also be combined with the direct wavefront sensing AO. The SIM is a method of making a light source illuminated on a sample into a pattern and then obtaining an image via computation. This method is attracting attention owing to the high resolution beyond the diffraction limit in live samples. However, there have been issues of the previous methods of SIM, e.g., the low signal-to-noise ratio (SNR), motion-induced artifacts occurred due to the characteristics of the in vivo sample. An optical-sectioning SIM integrated with direct wavefront sensing by the Shack-Hartmann sensor enabled to reduce the noise and correct the motion of sample [37]. Thus, three-dimensional images were obtained from mouse cortical neurons and zebrafish motor neurons in vivo. The widefield and optical sectioning SIM images of a Thy1-GFP mouse brain in vivo are shown in Fig. 8c. The left two panels are the widefield image with or without AO. Maximum intensity projections were acquired by a 10-μm-thick widefield. The right two panels are the optical sectioning SIM images with or without AO. The images of optical sectioning SIM were acquired using the z stack with a 0.5-μm step, 30-μm depth, 1024 × 1024 pixels at an 86-nm pixel size. The insets of maximum intensity projections are the zoomed-in images in the white boxes. The structures of the spine became clear acquired by the optical sectioning SIM as compared to the widefield owing to the effective reduction of out-of-focus background noise.

Another study reported that the method of widefield illumination, structured illumination, and confocal illumination was integrated into one system with AO, in which AO can be applied to direct or indirect wavefront sensing by simply changing the optical path [38]. This study implemented the all-in-one system combined with the image-based sensorless wavefront sensing, confocal sensorless adaptive optics, and direct wavefront sensing, thereby, developed the three-dimensional SIM. *C. elegans* in vivo images expressed by the adherens junction marker ajm-1::GFP are shown in Fig. 8d. The upper left panel is the overlay of the fluorescence image and a DIC phase image at the AJs, which are membrane-anchored structures stabilizing cellular contacts, in the posterior bulb of the pharynx. The fluorescent image was acquired by the confocal spot (inset). The lower left two panels are the widefield images of AJs after system correction and sample correction. The upper right four panels are three-dimensional SIM images of AJ 1 (inside red square) before and after correction. The lower right four panels are three-dimensional SIM images of AJ 2 (inside blue square) before and after correction.

### Indirect wavefront sensing for AO fluorescence imaging

The methodologies of indirect wavefront sensing are mainly classified to a modal method and zonal method. The modal method uses a continuous surface wavefront correcting instrument, and the wavefront is composed of the sum of the aberration modes. Individual aberration modes, for example, Zernike mode, are applied to the instrument. The images are quantified by metrics such as intensity, sharpness, or contrast. It is important to optimize the value of the metric in the limited time scale. For the zonal method, the wavefront is considered as the individual non-overlapping zone across the entire pupil. This method determines the slope of each area to correct the wavefront in the image shift at the focal point. Using a deformable mirror, each piston moved forward or backward to ensure that the rays are in-phase at focus. The segment is determined by strength measurements with different piston positions. In the Spatial Light Modulator (SLM), the wavefront correcting instrument is changed by the refractive index of the pixel.

**Fig. 9:**
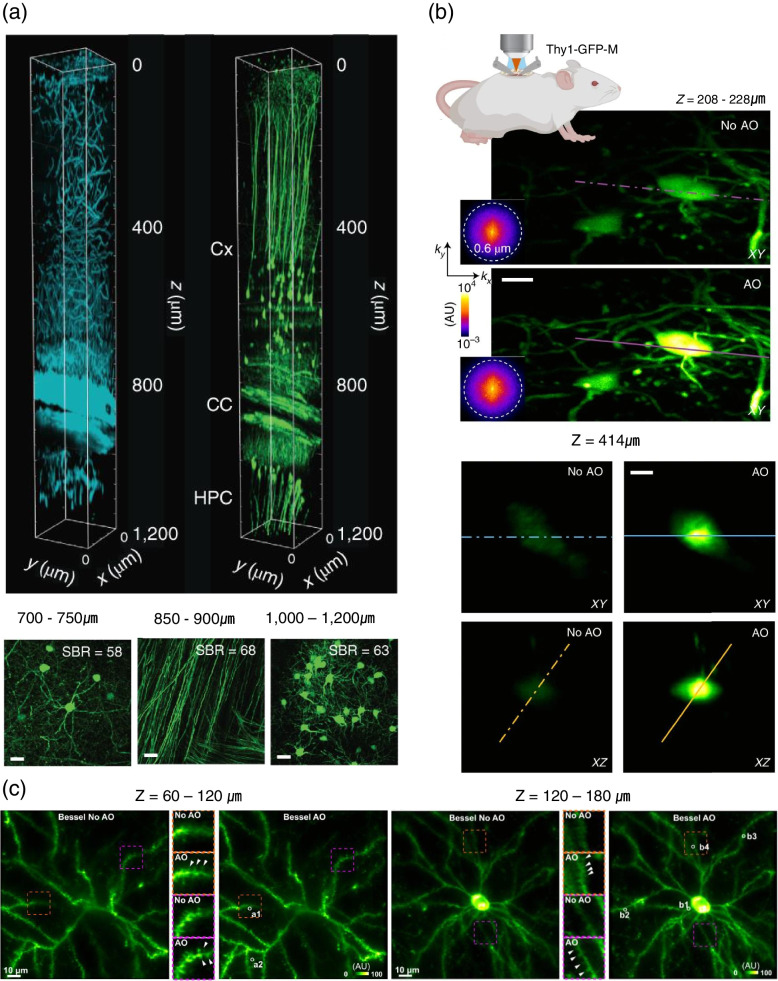
Indirect wavefront sensing with focus scanning microscopy. **a** The visual cortex and hippocampus at the depth-of-field 700–750 μm, 860–900 μm, and 1000–1200 μm for a EGFP–Thy1(M) mouse brain in vivo. Adapted with permission from Ref. [39]. **b** The synaptic structures in the deep cortical region of a mouse (Thy1-GFP-M) brain. Adapted with permission from Ref. [40]. **c** High and basal dendritic spines of a mouse V1 neuron in vivo. Adapted with permission from Ref. [41]. Scale bar in a, 20 μm. Scale bar in b is 10 μm

Imaging mammalian brain beyond the depth of 1 mm is typically difficult to achieve with a two-photon microscope, since out-of-focus background noise appears near the surface by the increased laser power. A three-photon laser scanning microscope can be an appropriate solution for this issue by integration with a modal-based sensorless wavefront sensing system. The mouse hippocampus at a depth of 1.4 mm was acquired by modal-based sensorless AO three-photon microscopy with active electrocardiogram gating [39]. It is also possible to perform calcium imaging in the deep-layer of astrocytes in a mouse brain. The laser at 1300 nm excitation wavelength was illuminated at the visual cortex and hippocampus in EGFP–Thy1(M) mouse brain. The left panel in Fig. 9a is the three-dimensional reconstruction image of three-photon image stack. The cyan indicates the third-harmonic signal, and the green indicates the GFP-labeled neurons. The right panel is the image of maximum intensity projection at several depths in the cortex (top), corpus callosum (middle), and CA1 of the hippocampus (bottom). In each image, the signal-to-background ratio (SBR) was evaluated at the corresponding depth.

The zonal method with frequency-multiplexed aberration measurement can be used to evaluate and correct the tissue-induced aberration [40]. The zonal indirect sensing AO two-photon and three-photon microscopes provided to measure the synaptic structures in the deep cortical region of mouse brain (Fig. 9b). The upper left panel shows the schematic diagram of the spinal cord in the mouse (Thy1-GFP-M) brain in vivo. The lower left panel is the image of maximum intensity projection of spinal cord neurons at 208–228 μm below dura without and with AO (phase modulation correction) with the excitation laser at the wavelength of 1300 nm. Insets show the spatial frequency of the corresponding fluorescence images. The right panel shows the image of a neuron in the lateral and axial direction, 414 μm below dura with and without AO (phase modulation correction).

Another study reported that the modal-based technique of indirect wavefront sensing combined with the Bessel beam was suitable for high-speed volumetric imaging [41]. The Bessel beam at the focal plane of objective lens was exploited in the two-photon microscope to measure and correct the sample-induced aberration, thereby, the large volume images at the high spatiotemporal resolution were obtained from the living zebrafish larval and mouse brains at the 500 μm of depth-of-field. The three-dimensional structures at high and basal dendritic spines of a mouse V1 neuron in vivo are shown in Fig. 9c. The field-of-view is 128 × 128 × 120 μm^3^ and covered both high and basal dendritic branches. The left panel shows images of high and basal dendritic branches with Bessel focus before and after aberration correction by indirect wavefront sensing at a depth of z = 60–120 μm. The right panel shows the images of high and basal dendritic branches at the depth of z = 120–180 μm. The inset is the zoomed-in image of the structure in the red dashed box. The white arrows indicate the spines resolvable after aberration correction only.

**Fig. 10:**
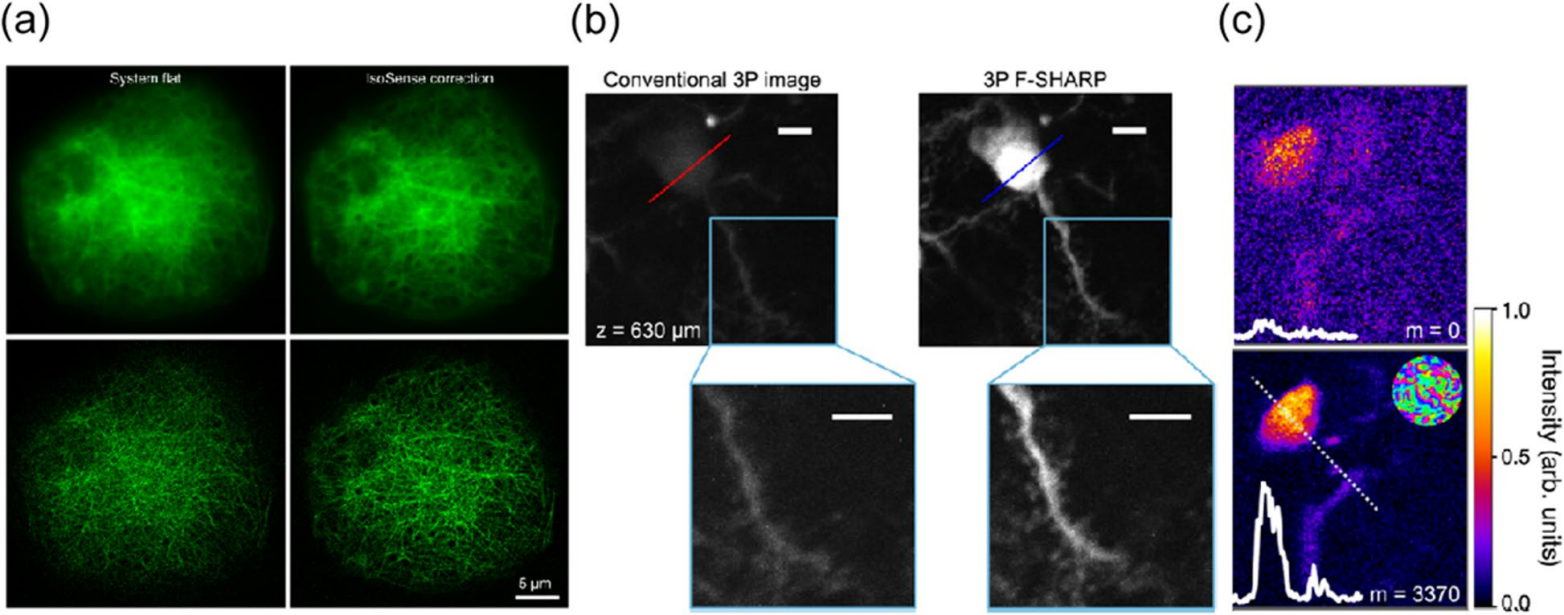
Indirect wavefront sensing with SIM and holographic method. **a** GFP expressed microtubule of Drosophila larval macrophage by SIM and IsoSense correction. Adapted with permission from Ref. [42]. **b** GFP-expressed pyramidal neuron below 630 μm in a living mouse brain measured by conventional and F-SHARP. Adapted with permission from Ref. [43]. **c** The single microglia cell from 530 μm thick hippocampus tissue of a mouse brain measured by conventional and DASH. Adapted with permission from Ref. [44]. Scale bar in b, 5 μm

Indirect wavefront sensing method can also be integrated with SIM [42]. In the image-based sensorless AO (IsoSense), Zernike polynomials were used as a standard basis for correction of sample-induced aberration. High spatial frequency content was calculated as a metric for the optimization to enhance the fine structure information of the sample. The green fluorescence protein (GFP) was expressed in the microtubule of Drosophila larval macrophage (Jupiter::GFP), and then super-resolved images were acquired by SIM combined with sensorless AO (Fig. 10a). The upper left panel is the microtubule image using system flat correction, and the upper right panel is the IsoSense corrected image. The lower two panels show the reconstructed image with SIM. The lower right panel indicates the benefit with IsoSense correction by SIM imaging.

The method of holographic scattering compensation is one way of indirect wavefront sensing. A study reported that the method is used for nonlinear excitation by two interference beams to measure the amplitude and phase of the scattered light within the tissue, and the information is used to correct the aberration (via focus scanning holographic aberration probing, F-SHARP) [43]. Thus, it is possible to acquire a high-speed image for the densely labeled sample with a three-photon laser, whereas it is typically difficult to obtain images with a two-photon laser. The images of the GFP-expressed pyramidal neuron below 630 μm in a living mouse brain were acquired by using a three-photon laser combined with F-SHRAP (Fig. 10b). The upper left panel is the maximum intensity projection by the conventional three-photon image with system correction only. The upper right panel is the corrected image obtained using the three-photon F-SHARP technique. The upper two images are stacked by 11 planes over a range of ±5 μm, and the isoplanatic patch size was about 80 μm. Insets of the lower two panels show zoomed-in images of dendrites and spines. The images were measured by the laser of 16 mW excitation power at the surface with a 1 MHz rate.

A different holographic technique of indirect sensing was reported, which provided non-invasive scattering compensation using the phase information obtained by a holographic phase interferometer, thereby, the system rapidly corrected the sample-induced aberration with just one measurement [44]. The dynamic adaptive scattering compensation holography (DASH) integrated with a two-photon microscope enabled to measure single microglia cell images from 530 μm thick tissue from the hippocampus of a mouse brain (Fig. 10c). The upper panel is the image of a microglia cell without correction. The lower panel is the corrected image by DASH after three iterative evaluations. The intensity profiles along the dashed line were extended over 20 μm as shown in the insets.

### Coherence-gated wavefront sensing and time-gated reflection matrix approaches

Most practical AO methods to date have been based on wavefront sensing using fluorescent signals. Direct wavefront sensing techniques require fluorescent beads or structures sparsely labeled with fluorescent dyes or proteins in tissues as guide stars. Indirect wavefront sensing approaches can be used for extended fluorescent targets, but generally require an iterative feedback control on a wavefront shaping device. These require a sufficiently bright fluorescent signal and numerous feedback iterations for successful closed-loop wavefront sensing and correction. During the iterative process, however, fluorophores are easily bleached due to repeated exposure of the sample to strong excitation light multiple times.

**Fig. 11:**
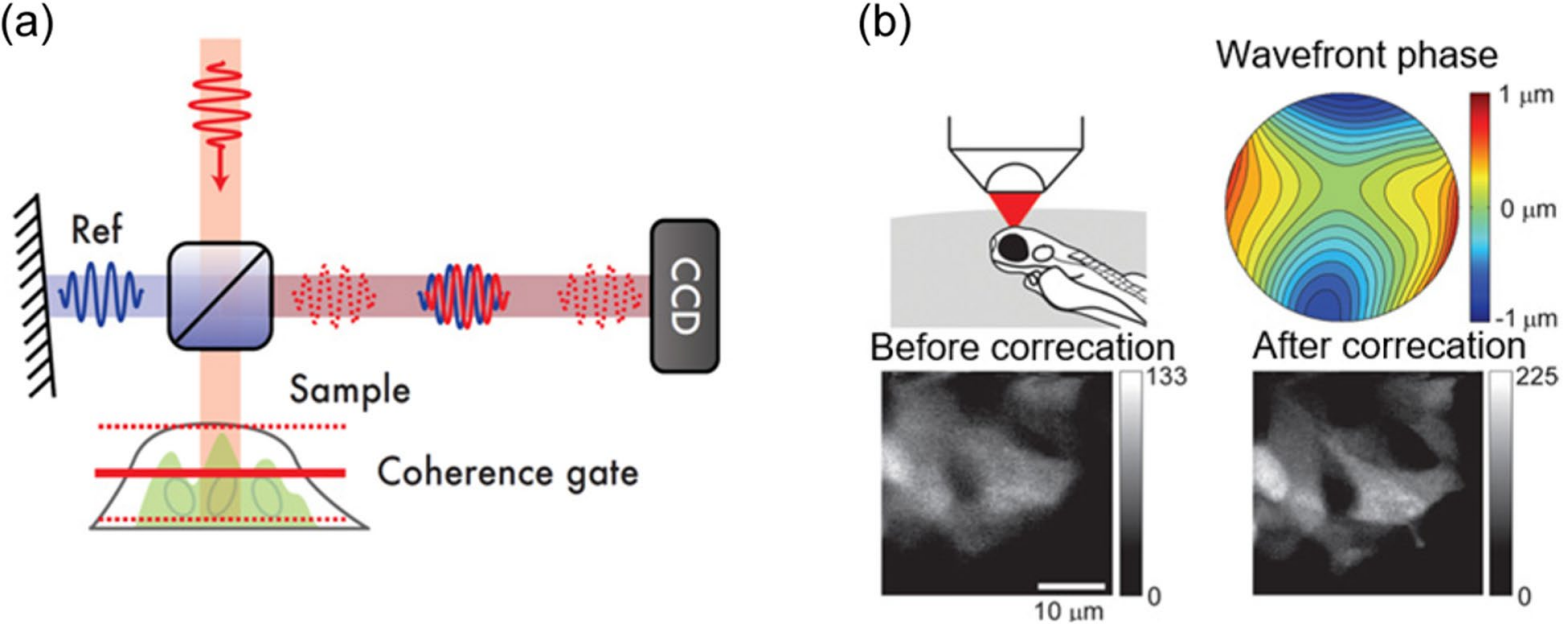
AO correction based on CGWS. **a** Schematics of low-coherence interferometer for CGWS. Adapted with permission from Ref. [77]. **b** CGWS-based AO imaging of the developing olfactory bulb in transgenic zebrafish larvae. Adapted with permission from Ref. [45]. Wavefront aberration map as measured by CGWS (top-right). Two-photon fluorescence images at a depth of 50 μm before aberration correction (bottom-left) and after aberration correction (bottom-right)

Another wavefront sensing strategy is coherence-gated wavefront sensing (CGWS) [45, 76], which measures the wavefront of elastically backscattered excitation light near the focus rather than fluorescent light. In thick, highly scattering samples, such as biological tissues, most of the backscattered light originates from multiple scattering outside of the focal plane of interest. These multiply scattered photons do not provide wavefront aberration information about the focal point. Thus, it is necessary to effectively discriminate ballistic light scattered from the focal region from the predominant multiply scattered photons. The depth-resolved wavefront sensing on elastically backscattered light can be performed with low coherence interferometry. The two configurations, phase-shifting interferometry [76] and digital off-axis holographic interferometry [77] are most widely used. Therein, an interferogram formed by the backscattered wave from the sample and a reference planewave with a low coherence light source are measured using a camera (Fig. 11a). The amplitude and phase images of the backscattered wave field are obtained by processing the measured interferogram. The interference signal only appears when the sample and reference arms are matched within the coherence length of the light source, which leads to depth discrimination. Due to the extremely high detection sensitivity of interferometric detection, the CGW-based AO methods allow the use of considerably lower laser power compared to fluorescence-based wavefront sensing AOs. Therefore, it is highly desirable for samples that are easily bleached or weakly fluorescent, and it can even be used for completely nonfluorescent samples.

CGWS-based wavefront sensing and correction was applied to two-photon fluorescence imaging in a living animal [45] (Fig. 11b). In this study, the sample-induced wavefront aberration of the coherence-gated sample light was obtained from interferograms recorded for 20 slightly different focus positions. The first 28 Zernike modes were used to compensate for the measured wavefront aberration. The shape of the deformable mirror by which the incident two-photon excitation laser beam is reflected was changed for wavefront correction. The intensity and contrast of two-photon fluorescence signals were significantly improved with the wavefront correction compared to before correction.

To successfully extend CGWS-based AO to deep imaging in strongly scattering samples, there are two major challenges that need to be addressed. The first is the efficient removal of strong background noise caused by multiply scattered light, which cannot be rejected by coherence gating with a finite coherence length. The second is to retrieve wavefront aberration information for the input path from the wavefront of the backscattered wave measured in an epi-detection geometry. The backscattered wave experiences wavefront distortion twice as it propagates through the sample in incoming and outgoing directions. Moreover, there is usually no bright point-like reflectors that can serve as guides at a target depth. In most cases, light is backscattered by random scatterers, resulting in nearly speckle-like patterns in the measured wavefronts. This situation makes it difficult to extract wavefront distortion in only the incoming path from the measured wavefront.

**Fig. 12:**
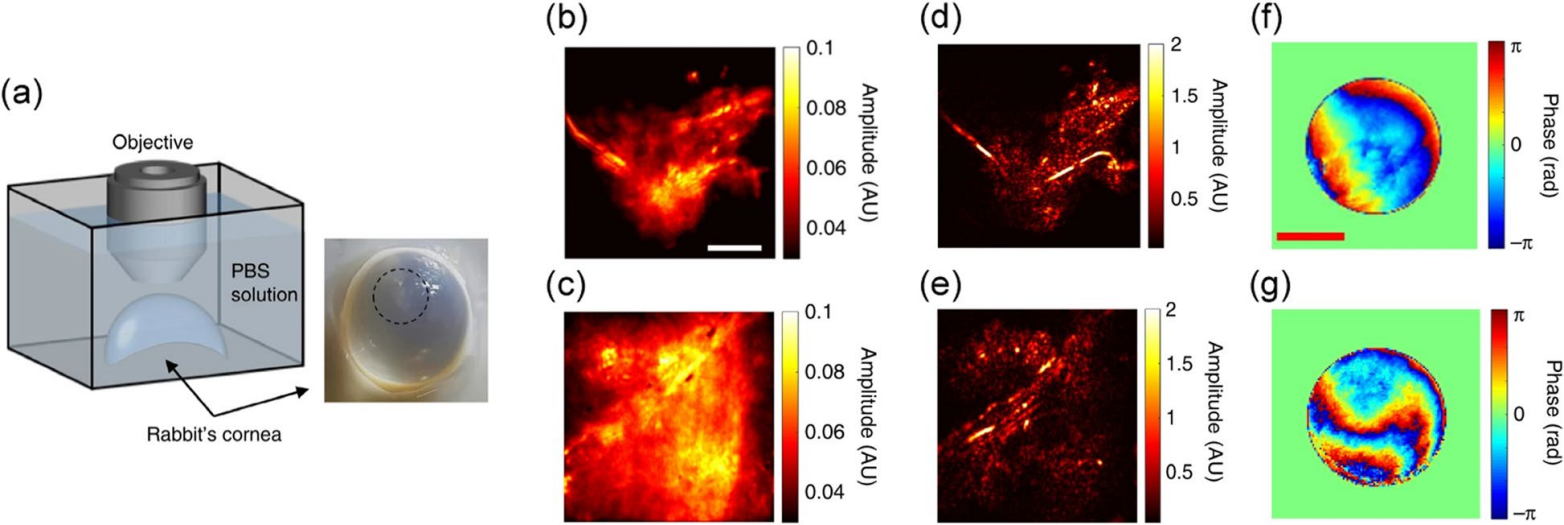
CLASS microscopy for imaging the hyphae of Aspergillus cells in a rabbit’s cornea. **a** Layout of imaging geometry. **b**,**c** Incoherent addition of time-gated reflection images. **d-i**, Same as (**b**,**c**) respectively, after aberration correction by CLASS algorithm. **f**,**g** Identified sample-induced wavefront aberrations for input and output directions, respectively. Adapted with permission from Ref. [46]

A technique termed closed-loop accumulation of single scattering (CLASS) microscopy [46] has been proposed to separately identify one-way aberrations from highly distorted wavefronts of double-passed waves. To do this, CLASS microscopy measures a time-gated reflection matrix [78] composed of a set of coherence-gated electric-field images of the backscattered waves recorded for all possible illumination field modes. The reflection matrix is the transfer function matrix of the imaging system including the sample, which directly relates the waves at the input and output. Then, the input and output wavefront aberrations are identified computationally by applying a unique aberration correction algorithm to the measured reflection matrix. The key idea of the correction algorithm is to jointly find the wavefront aberrations and the object image at the focal plane in such a way that the total intensity of the reconstructed object’s image is maximized. The hyphae of Aspergillus cells deep inside an ex vivo fungal keratitis-affected rabbit’s cornea were imaged by the CLASS microscopy (Fig. 12). To construct a time-gated reflection matrix, the incident angle of planewave illumination was scanned, and the amplitude and phase maps of backscattered waves from the sample were recorded with digital off-axis holographic interferometry. In the image post-processing, angle-dependent phase retardations for both the input and output waves were identified (Fig. 12f and g), and the aberration-corrected image was simultaneously reconstructed (Fig. 12d and e). Although the infected cornea is highly turbid because the fungi act as scattering particles, the CLASS algorithm enabled near-diffraction-limited imaging of fine structures of hyphae. A spatial resolution of 600 nm was observed up to the imaging depth of seven scattering mean free paths.

**Fig. 13:**
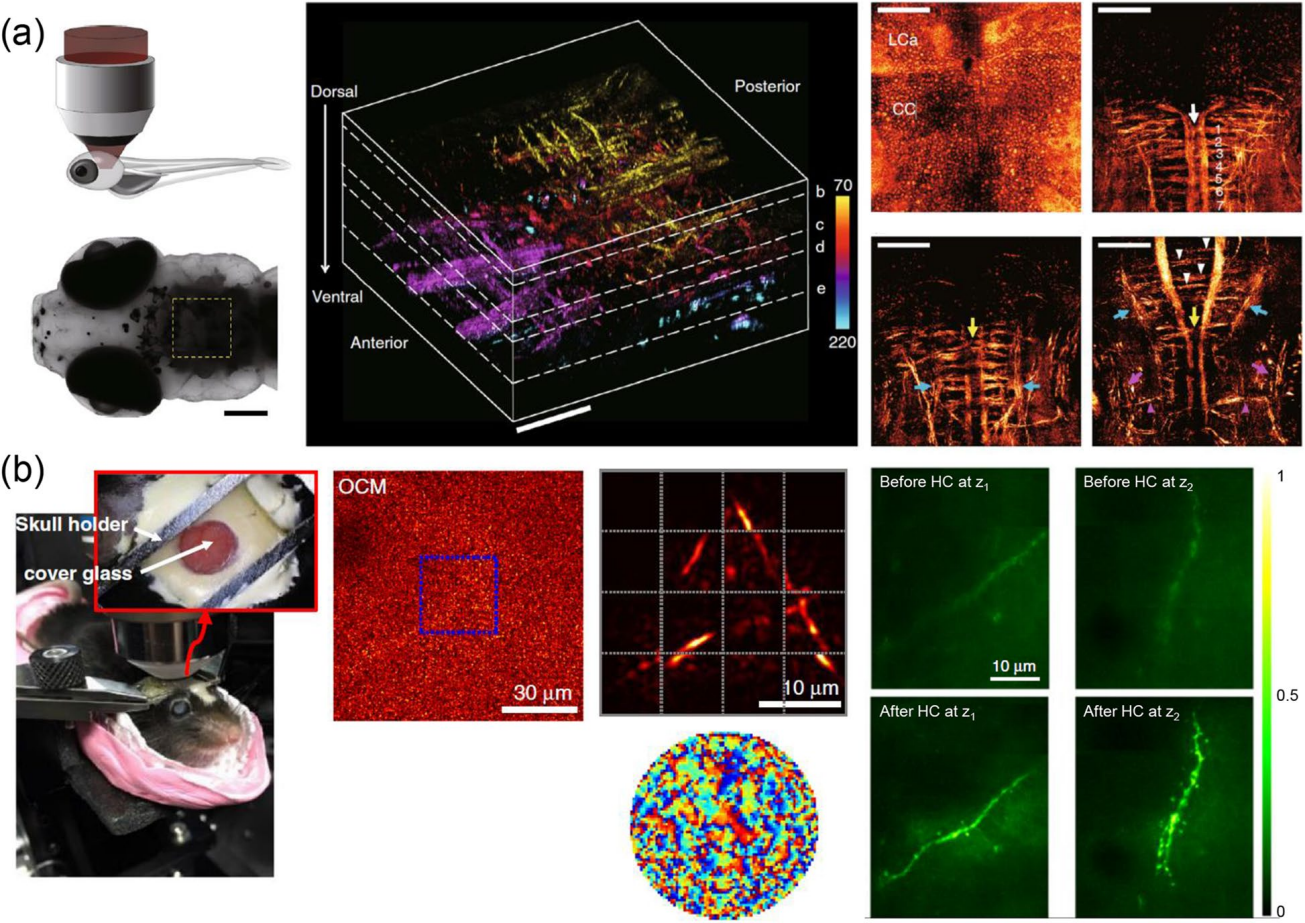
High-speed time-gated reflection matrix approaches for in vivo imaging. **a** Label-free volumetric images of the hindbrain of a 10-dpf larval zebrafish taken by AO-SASM. Adapted with permission from Ref [47]. **b** In vivo through-skull mouse brain imaging with LS-RMM. Adapted with permission from Ref. [48]. OCM: in vivo through-skull optical coherence microscopy image. LS-RMM: LS-RMM image corresponding to OCM image. Before HC: ex vivo through-skull two-photon images showing neuronal dendrites in the brain of a Thy1-EGFP line M mouse before hardware wavefront correction. After HC: two-photon images after hardware wavefront correction, corresponding to before HC images. Imaging depths: z_1_ = 113 ± 1.5 μm, z_2_ = 122 ± 1.5 μm. Adapted with permission from Ref. [48]

In the first implementation of the CLASS microscope, a slow liquid-crystal spatial light modulator (LCSLM) was used to scan the angle of illumination planewave, which limits the applications of imaging in living specimens. Thereafter, the high-speed image acquisition technique was developed to perform in vivo neuroimaging by synchronously scanning the angle of the sample and reference waves using fast galvanometer mirrors to measure the time-gated reflection matrix (adaptive optical synchronous angular scanning microscope, AO-SASM) [47]. With the advantage of high-throughput image acquisition, the acquisition time for single-depth image was reduced from a few minutes to 0.22 s. Figure 13a shows label-free volumetric imaging for the hindbrain of a living zebrafish taken by AO-SASM. Fine axon branches of reticulospinal neurons and torus semi-circularis in the neural network were clearly visualized with a near diffraction-limit spatial resolution of 370–480 nm up to a depth of 220 μm.

Recently, another study reported that the time-gated reflection matrix approach combined with a two-photon fluorescence microscope, which is referred to as laser-scanning reflection matrix microscopy (LS-RMM) [48], was demonstrated for two-photon fluorescence imaging the brain of an intact living mouse skull (Fig. 13b). The mouse skull is composed of several layers of microstructures, causing extreme, high-order aberrations and a very small isoplanatic patch size, which is defined by the field of view within which the wavefront distortions are closely correlated. First, optical coherence imaging was performed to obtain information about skull-induced anisoplanatic aberrations over a large field of view. To do this, the time-gated reflection matrix was measured in position space by scanning a focused excitation laser beam over a specific field of view instead of scanning the angle of planewave. The field of view was then divided into several subregions, and the AO algorithm based on CLASS was applied to each subregion to obtain local aberrations. For aberration correction, the phase conjugations of the identified local aberration maps were sequentially applied to a LCSLM introduced in the excitation laser beam path. With this hardware aberration correction applied, the two-photon excitation laser beam incident through the skull was focused on a tight spot in the brain, increasing the fluorescence signal intensity by up to 19 times. The aberration-corrected two-photon fluorescence images shown in Fig. 13b clearly showed the structure of neuronal dendrites and dendritic spines with near-diffraction-limited resolution of 380 nm.

## Conclusion and outlook

In this review, we have provided broad discussion of deep-tissue imaging encompassing the fields of physics, chemistry, biology, engineering, and medicine. We investigated the major issue that optical imaging deeper into a thick tissue specimen leads to increased multiple light scattering and complex wavefront aberrations. The intensities of ballistic and forward multiply-scattered waves, which are used for wavefront correction and image information delivery, decay exponentially with imaging depth. We surveyed the diverse methodologies employed to solve the problems of wave distortion and signal attenuation including multi-scale imaging with respect to macro-mesoscopic and microscopic regimes.

We presented imaging probes as macroscopic or mesoscopic imaging approaches that are readily applicable for translational medicine and clinical studies in Section 2. Such studies are categorized into two strategies. First, using the long wavelength is advantageous for tissue penetration (NIR-II imaging) due to its long scattering mean free path of the tissue. Secondly, using lights without excitation to minimize background signal (bioluminescence, chemiluminescence, and afterglow imaging) for macro-mesoscopic imaging is useful since wave diffusion of the excitation path does not need to be considered even if visible light is used for emission. It is obvious that the NIR-II region is more suitable for deep tissue imaging than other regions of light. However, there have been few imaging probes generating NIR-II light, and none of them are approved for human trials. This is partially because the chemical structures of small molecule dyes with NIR-II region are extremely hydrophobic, so that it is hard to make them in an aqueous solution form for injection using a syringe. Therefore, developing new probes with long wavelengths and lower hydrophobicity is essential. Bioluminescence imaging uses luciferase proteins, which can be degraded by various enzymes in the human body. In addition, it needs two components, luciferase and its substrate (like luciferin or CTZ), making this strategy different from other optical imaging using one material. Chemiluminescence is useful in that it is possible to develop rationally designed probes in response to various kinds of molecules in the body. However, in both bioluminescence and chemiluminescence, the imaging signals change with time, which is a big hurdle to quantifying the signals for diagnosis of disease. The time-dependent change of signals is a more serious problem in afterglow imaging. Afterglow imaging has the advantage of generating background signal-free light generation with relatively simple components than other imaging probes, but its imaging signal intrinsically decreases during imaging. Therefore, it would be important to extend the duration of imaging signals and provide sufficient time for imaging with minimal signal changes.

We presented the optical techniques based on AO as microscopic and mesoscopic imaging approaches, which are more suitable for biomedical and nanophotonic applications, in Section 3. These involve direct and indirect sensing based on florescent light, coherence-gated direct wavefront sensing, and time-gated reflection-matrix approaches. The optical techniques can be beneficial for observing organoids with human-derived induced pluripotent stem cells (iPSCs) to understand Alzheimer’s disease as a study of translational medicine [79]. The optical techniques can also be beneficial to investigate a small diagnostic region of interest deep in tissue with subcellular resolution, in particular to detect early stages of skin cancer for clinical applications. Despite there are several clinical imaging techniques available for detecting melanoma, their spatial resolutions are not high enough to diagnose early stages of skin cancer [80]. A non-toxic fluorescent dye that can be excited in the NIR-II wavelength region is a prerequisite for the use of AO techniques based on fluorescent light in deep-tissue clinical imaging applications. AO approaches based on time-gated reflection matrix are emerging as an invaluable imaging strategy for label-free, non-invasive high-resolution clinical diagnostics, such as screening for skin cancers at an early stage.

The optical imaging depths achievable with current AO methods are limited to a few hundred microns in biological tissues. Extending the imaging depth by one scattering mean free path of a specimen would therefore require an order of magnitude or more improvement in the performance of wavefront correction. First, increases in the number of spatial modes for wavefront correction need to be achieved for deeper imaging depth than the state of the art in current AO methods. Most AOs have so far dealt with low spatial frequency aberrations, a smooth wavefront phase variation in the pupil plane. This is mainly due to the limitation of the number of actuators in wavefront control devices and is typically in the range of several tens to hundreds. The ability to measure and control a larger number of spatial modes (> 1000) in a wavefront will be important for resolving more complex aberrations or for wavefront distortions involving multiple scatterings in current AO technologies. Second, it needs to be able to cope with spatially varying aberrations, which are mainly caused by three-dimensional variations in the refractive index inside a volumetric specimen. Most AO methods developed so far deal with pupil aberrations that cannot adequately represent such volumetric specimen-induced aberrations. Although a complex wavefront correction is possible with a large number of correction modes, it is only applicable to a small isoplanatic patch, that is, the limited field region where the wavefront distortions are invariant. A different correction could be applied independently to each isoplanatic patch. However, as the imaging depth becomes deeper, the size of isoplanatic patch becomes smaller. This divide-and-conquer method is no longer practically applicable. A technique called multi-conjugate AO [81–83] may be a possible solution to overcoming this issue in deep-tissue imaging. To compensate for wavefront aberrations induced by a volumetric specimen, the multi-conjugate AO utilizes more than one wavefront correction device, each placed in a plane optically conjugated to a depth of the specimen. Finally, it is necessary to develop advanced AO techniques that can efficiently control higher-order multiple scattering for more dramatic improvements in imaging depth. It is generally accepted that focusing light beyond one transport mean free path (~ 1 mm in biological tissues), i.e., where the propagation direction of waves has been totally scrambled, is infeasible [3, 7]. Until now, AO methods have primarily relied on the use of singly-scattered and forward multiply-scattered waves to focus light or inside a specimen. Utilizing multiple scattering to focus light will be an important in future AO technologies that break optical imaging depth limits set by the transport mean free path.

The short penetration depth is an intrinsic limitation of optical imaging. It is obvious that the optical probes and techniques could not remove this limitation completely, but relatively increase the penetration depth compared to traditional materials and methods. It was reported that the penetration depth of the representative conventional optical probe, indocyanine green is about 4 mm [84]. Some of the introduced optical probes showed better data, but they were still below 20 mm. However, when we imagine the particular situation in clinic such as image-guided surgery of cancers after laparotomy, relatively increased penetration depth of imaging may enable more accurate and successful removal of the buried cancer tissue. In both imaging probes and optical techniques, there is a gap between research in labs and clinical applications. Imaging probes need to be injected into human body, so their biocompatibility must be confirmed. Most of the injected probes are expected to be secreted from body by urine or feces after several hours or days. However, it would be important to guarantee their complete secretion by data for further application, and hydrophilicity and small size would be beneficial to be secreted quickly. New optical techniques generally use complicate devices, and their accessibility to disease sites in body needs to be considered carefully. The developed probes and devices should be approved by the FDA for human applications, and the related processes could be long and require a lot of efforts and funding. Therefore, cost and expected market size would be important factors. Human applications of optical imaging have been restricted to date, and difficulties associated with deep tissue penetration present the most serious obstacles. Researchers have been working for long time to overcome these problems, and recent advances in both imaging probes and optical techniques showed potential. Therefore, we expect that the role of optical imaging will become even greater in the diagnosis of disease in the future.

## Data Availability

Not applicable because this is a review article and no data were newly generated.
